# Gp41 dynamically interacts with the TCR in the immune synapse and promotes early T cell activation

**DOI:** 10.1038/s41598-018-28114-5

**Published:** 2018-06-27

**Authors:** Oren Yakovian, Roland Schwarzer, Julia Sajman, Yair Neve-Oz, Yair Razvag, Andreas Herrmann, Eilon Sherman

**Affiliations:** 1 Racah Institute of Physics, The Hebrew University, Jerusalem, 91904 Israel; 2 Department of Biology, Molecular Biophysics, Humboldt Universität zu Berlin, 10115 Berlin, Germany

## Abstract

The HIV-1 glycoprotein gp41 critically mediates CD4^+^ T-cell infection by HIV-1 during viral entry, assembly, and release. Although multiple immune-regulatory activities of gp41 have been reported, the underlying mechanisms of these activities remain poorly understood. Here we employed multi-colour single molecule localization microscopy (SMLM) to resolve interactions of gp41 proteins with cellular proteins at the plasma membrane (PM) of fixed and live CD4^+^ T-cells with resolution of ~20–30 nm. We observed that gp41 clusters dynamically associated with the T cell antigen receptor (TCR) at the immune synapse upon TCR stimulation. This interaction, confirmed by FRET, depended on the virus clone, was reduced by the gp41 ectodomain in tight contacts, and was completely abrogated by mutation of the gp41 transmembrane domain. Strikingly, gp41 preferentially colocalized with phosphorylated TCRs at the PM of activated T-cells and promoted TCR phosphorylation. Gp41 expression also resulted in enhanced CD69 upregulation, and in massive cell death after 24–48 hrs. Our results shed new light on HIV-1 assembly mechanisms at the PM of host T-cells and its impact on TCR stimulation.

## Introduction

Viruses interact with a manifold of host cell components in order to facilitate different steps of the viral life cycle. The envelope protein (Env) of HIV-1 has been shown to mediate host cell binding and the fusion between cellular and viral membrane. The functional form of the Env spikes is composed of trimers of non-covalent gp120-gp41 heterodimers. Whereas the surface subunit gp120 initiates cell infection by binding the primary receptor CD4 and the co-receptor CCR5 or CXCR4, the membrane spanning subunit gp41 permits target membrane penetration and fusion^1^.

Viral assembly and budding depends on gp41 aggregation at the plasma membrane (PM)^2^. This assembly process is regulated by both viral and cellular factors^3^, however it is yet unclear what mechanism is enabling efficient interactions between the viral structural proteins on the host cell surface in the final stages of virus genesis^3^. Specifically, it has been shown that gp41 interacts with specific domains of the T cell antigen receptor (TCR)^4^, and that this association can exert immunosuppressive effects^5^, e.g. by interrupting with the TCR complex and its function^6^.

Although critical to viral budding and infection, the process of gp41 clustering and dynamic organization at the PM of infected cells and its effect on TCR signalling upon T cell activation remain poorly understood. A critical limitation of studying viral assembly concerns the small, nanometer sized nanoclusters of viral and cellular proteins that are involved in this process^7^. This prevents the study of viral assembly in intact cells using diffraction-limited light microscopy. Some recent studies have turned to super-resolution microscopy for that task^8–11^. Specifically, single molecule localization microscopy enables the study of protein assembly at the PM of intact cells in single molecule detail with resolution down to ~20–30 nm (Fig. S1A)^12,13^.

In order to understand the assembly mechanisms of gp41 at the PM of intact host T cells in molecular detail, we employed here photoactivated localization microscopy (PALM)^12^ of individual gp41 proteins at the PM of fixed and live cells. We further employed PALM in two-colours to resolve the interactions of gp41 and its mutants (Fig. S1B,C) with cellular proteins. Following previous studies that focused on gp41-derived peptides and their interaction with the TCR^5,6^, we studied the interaction of full-length and truncated gp41 with the TCR. We found that the transmembrane domain (TMD) of gp41 mediates its interaction with the TCR at the PM of activated and non-activated T cells. This interaction, confirmed by FRET, depended on the virus clone, was reduced by the gp41 ectodomain in tight contacts of the cell, and was completely abrogated by mutation of the gp41 transmembrane domain. Strikingly, gp41 preferentially colocalized with phosphorylated TCRs at the PM of activated T-cells and promoted TCR phosphorylation. Gp41 expression also resulted in enhanced CD69 upregulation, and in massive cell death after 24–48 hrs. Our results shed new light on the assembly mechanism of gp41 at the PM of T cells and may indicate new ways for intervening with T cell signalling, viral budding and repeated HIV-1 infection.

## Results

### Description of gp41 and its variants used in the study

The gp41 subunit is composed of ~345 aa, and contains various domains (see Fig. S1B). The ectodomain (ED) consists of several functionally, highly relevant motifs. The N terminal motif of the ED, named fusion peptide (FP), penetrates the host cell membrane and induces membrane fusion. Two next motifs are the N and C terminal heptad repeats (N-HR & C-HR). These hydrophobic sequences are critical to membrane merging and fusion pore formation^14^. The membrane proximal tryptophan rich ED region (MPER) is required for virus infectivity^15,16^. MPER contains a Galactosyl Ceramide (GalCer) binding site and the Cholesterol Recognition Amino acid Consensus (CRAC) domain, which are involved in HIV transcytosis and gp41 lateral sorting, respectively^17^. The ED is followed by a transmembrane domain (TMD). The cytoplasmic tail of gp41 (CT) is composed of three LLP (lentivirus lytic peptides) motifs. This part is involved in viral maturation and gp41-gp120 heterodimer stability^18^. In this work, constructs of gp41 derived from the primary isolate JRFL^19^ or the lab adapted HXB2 strain contained the LLP, TMD and MPER motifs. Throughout this study, we marked these constructs as gp41(JRFL/HXB2)ΔED* for simplicity, as they lack most of the ED. Full length constructs of gp41(HXB2) included the entire ED (i.e. MPER, C-HR, N-HR and FP domains). All constructs were tagged by either PAGFP, Dronpa or PAmCherry photoactivatable fluorescent proteins (PAFPs) for PALM imaging, and are further discussed in the following sections.

### Gp41 interacts with the TCR at the PM of CD4^+^ T cells

**Figure 1:**
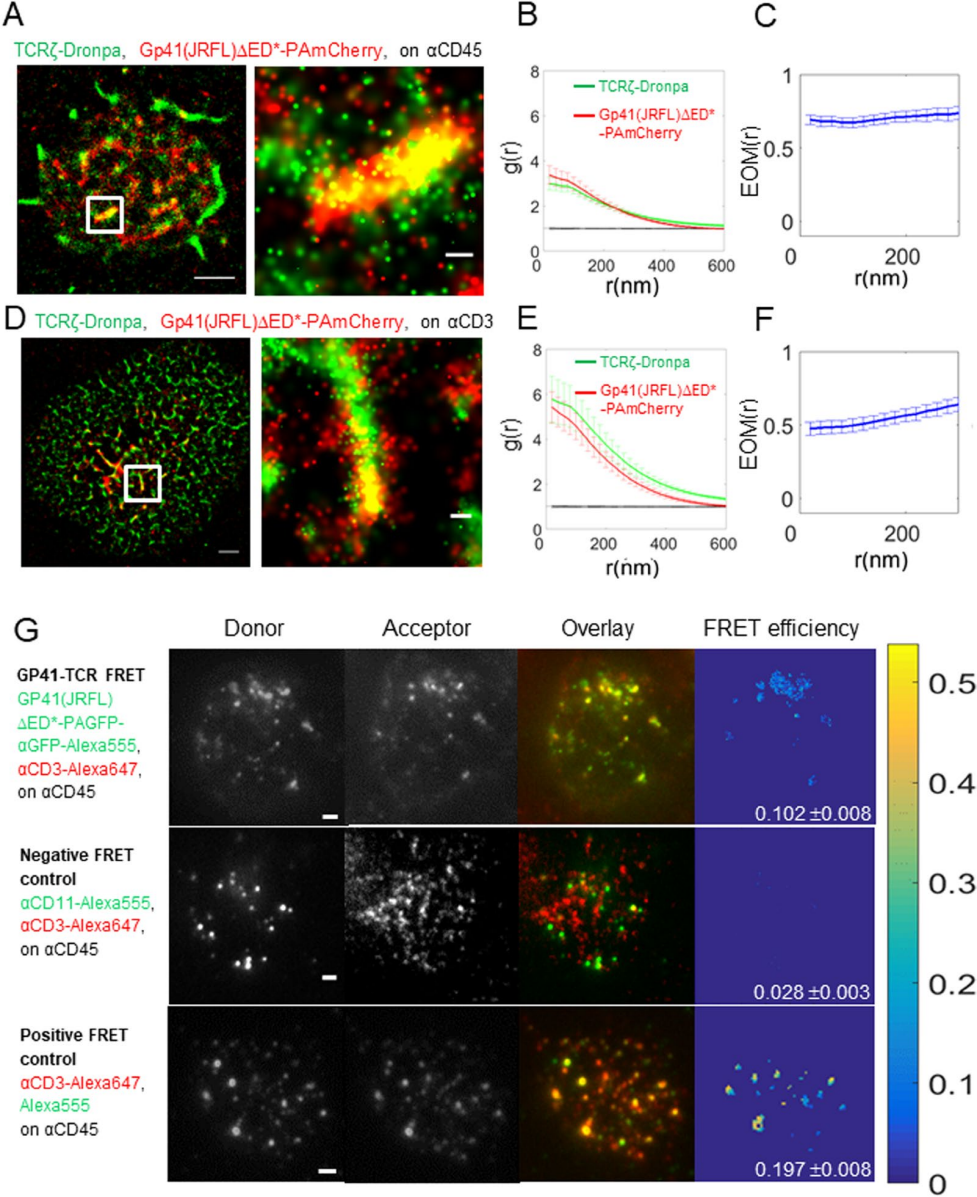
Gp41 and TCR closely associate at the PM of CD4^+^ T cells. (**A**) Two-colour PALM imaging of fixed E6.1 Jurkat cells expressing TCRζ-Dronpa and gp41(JRFL)ΔED*-PAmCherry (simply notated gp4-PAmCherry below) on an αCD45-coated coverslips. Cells were dropped and let spread on the coverslip for 3 min before fixation. Shown is a representative cell (N = 20). Bars – 2 μm (left) and 200 nm (right). (**B**) PCF of TCRζ-Dronpa (green) and gp41-PAmCherry (red). (**C**) EOM of TCRζ-Dronpa and gp41(JRFL)ΔED*-PAmCherry. (**D**) Two-colour PALM imaging of fixed E6.1 Jurkat cells expressing TCRζ-Dronpa and gp41(JRFL)ΔED*-PAmCherry on a TCR stimulating, αCD3-coated coverslips for 3 min before fixation. (N = 13). (**E**) PCF of TCRζ-Dronpa (green) and gp41(JRFL)ΔED*-PAmCherry (red) on stimulatory coverslips. (**F**) EOM of TCRζ-Dronpa and gp41(JRFL)ΔED*-PAmCherry. Error bars are SEM. (**G**) (top row) FRET imaging of fixed E6.1 Jurkat cells expressing gp41(JRFL)ΔED*-PAGFP on an αCD45-coated coverslips. TCRs were stained using αCD3ε-Alexa647 primary antibody. Gp41(JRFL)ΔED*-PAGFP molecules were stained using αGFP-Alexa555. Cells were dropped and let spread on the coverslip for 3 min before fixation. Shown is a representative cell (N = 14). Bars – 2 μm. (middle row) FRET imaging of fixed E6.1 Jurkat cells on an αCD45-coated coverslips, as a negative control to the results in the upper row. TCRs were stained using αCD3ε-Alexa647 primary antibody. CD11 molecules were stained using an αCD11-Alexa555 primary antibody. Cells were dropped and let spread on the coverslip for 3 min before fixation. Shown is a representative cell (N = 12). Bars – 2 μm. (bottom row) FRET imaging of fixed E6.1 Jurkat cells on an αCD45-coated coverslips, as a positive control to the results in the top row. TCRs were stained using αCD3ε-Alexa647 primary antibody and a secondary antibody carrying Alexa555 that targeted the αCD3ε-Alexa647 primary antibody. Cells were dropped and let spread on the coverslip for 3 min before fixation. Shown is a representative cell (N = 13). Bars – 2 μm. Colour bar – FRET efficiency.

During virus entry, the gp120-gp41 complex interacts with the co-receptors CD4 and CCR5 or CXCR4 to facilitate the fusion of viral and host membranes. It has also been reported to directly interact with a plethora of other cellular proteins^20^. The biological relevance and function of most of these interactions are currently not known. In recent years, the Shai group has shown that gp41-derived peptides are able to insert into the PM of CD4^+^ T cells, interact with the TCR and interfere with TCR activation^5,6^. This interaction might affect cell activity during both entry, assembly and budding of the virus at the PM of host cells. Here, we turned to the expression of gp41 in intact cells, and the imaging of its association with the TCR in single molecule detail using two-colour PALM. The TCR complex was imaged using TCRζ labelling with the PAFP Dronpa (TCRζ-Dronpa)^21^. Gp41 variants were tagged with PAmCherry^22^ (Fig. S1A,B). We started by imaging these two proteins in cells adhering to coverslips coated with an anti-CD45 antibody, that does not directly stimulate the TCR. These coverslips cause cell attachment and spreading, thus enabling cell imaging in total internal reflection (TIRF) mode, without direct TCR stimulation (Fig. 1A). We dropped cells onto the coverslips and let them attach the surface for 3 min before fixing and imaging. We observed that both TCRζ and gp41(JRFL)ΔED* (see Fig. S1A) significantly self-associated, thus forming self-clusters, as their univariate pair-correlation function (PCF) was up to 3–4 times higher than 1, the expected value for a Poisson random distribution (Fig. 1B). We further noted that TCRζ and gp41(JRFL)ΔED* closely associated in these clusters, as their self-clusters often overlapped (Fig. 1A). To quantify this association for multiple cells, we employed here the following extensions of previously used second-order statistics that we term the extent of mixing (EOM)^13,23^ and the standardized bivariate PCF (SBPCF; see further details in Fig. S2A and in the Analyses part of the SI). The EOM captures the extent of the molecular interaction in a normalized range between 0 (no interaction) and 1 (strong, homogeneous interaction). Previously, we have shown that bivariate PCF (BPCF) and EOM can capture different extents of protein-protein interactions in single molecule localization microscopy (SMLM) images^13,23^. We have further shown that BPSF statistics (and likewise, EOM) are largely insensitive to multiple artefacts related to SMLM, including the relative molecular counts of the interacting species and their localization uncertainty^13^. Here, we present these published statistics with a few changes that allow for more reliable quantification of protein-protein interactions for multiple cells. See definitions and further details in the Analyses parts of the Methods and SI.

Returning to the association between gp41(JRFL)ΔED* and TCRζ on αCD45-cotated coverslips, we note that their EOM takes a value of ~0.7 (Fig. 1C). Their SBPCF lies below the ‘Random Labelling’ (RL) model, through which the labels of proteins are randomized while their detected positions are kept. This model indicates a strong, homogeneous interaction (Fig. S2B). The measured SBPCF also lies above the no-interaction (NI) model, through which all protein positions are completely randomized (Fig. S2B). Thus, our results indicate significant interaction of gp41(JRFL)ΔED* and TCRζ, as identified in the PALM images (Fig. 1A). We further tested this association under another non-stimulating condition, by imaging cells dropped on αCD11-coated coverslips (Fig. S3A). We found similar self-clustering of both TCRζ and gp41(JRFL)ΔED* (Fig. S3B), and significant co-clustering (albeit, somewhat reduced in comparison to results on αCD45; Fig. S3C).

A similar association was found for cells on TCR-stimulating coverslips by coating with the αCD3ε antibody (Fig. 1D). As expected from previous studies^23–25^, we note from the PCF statistics that TCR self-clustering was enhanced in comparison to non-stimulating conditions (compare Fig. 1E,B; see also Fig. S3K for a comparison with TCR self-clustering in cells without gp41). However, here we note that also gp41(JRFL)ΔED* self-clustering grew upon T cell activation, by a factor of ~2. Thus, T cell activation promotes gp41(JRFL)ΔED* clustering. The EOM showed a value of 0.5–0.6 and the SBPCF curves were between the RL model and the NI model, indicating significant interaction between gp41(JRFL)ΔED* and TCRζ (Fig. S2B,C). Thus, gp41(JRFL)ΔED* and TCRζ significantly interacted under both TCR stimulating and non-stimulating conditions. To further test the validity of our conclusions regarding the interaction of the TCR with gp41(JRFL)ΔED*, we conducted 2-colour PALM imaging and analyses of gp41(JRFL)ΔED* and an unrelated self-clustered membrane protein (Syntaxin1A). As expected, these proteins failed to show a significant interaction (Fig. S3D–J), thus further confirming our conclusion about the interaction of TCR and gp41(JRFL)ΔED*. (See further details regarding controls for our PALM imaging and statistical analyses in the section on Materials and Methods below and in the SI).

PALM imaging and second-orders statistics indicate the non-random statistical interaction of gp41(JRFL)ΔED* and the TCR at the PM of T cells. However, PALM resolution is limited to ~20 nm and thus cannot report on direct association of the two proteins. Previous biochemical and biophysical results have been used to report on the association between gp41 derived peptides inserted into the PM of T cells and the TCR^5,6^. However, this interaction has not been reported for intact proteins or found by other techniques^20^. To study the physical interaction of gp41 and TCR, we turned to Förster Resonance Energy Transfer (FRET) imaging of the two proteins. FRET reports on the proximity between a Donor and an Acceptor fluorophores in a range between 2 and 10 nm, and thus has been widely used to report on physical proximity and association of two labelled proteins^26,27^. Here, we employed the commonly used approach of sensitized emission for FRET measurements^20,21^. Specifically, we targeted surface exposed proteins of live Jurkat E6.1 cells by labelling the TCR with an αCD3ε-Alexa647 primary antibody and gp41(JRFL)ΔED*-PAGFP with an αGFP-Alexa555 primary antibody. We measured an average FRET level of 10.2 ± 0.8% for N = 14 cells (Fig. 1G). This level of FRET is indicative of a direct interaction between the proteins (proximity of less than 10 nm, considering the size of the antibody stains). To verify the significance of this interaction, we compared the results to a negative FRET sample that checked the interaction between the TCR (CD3ε) and CD45 in Jurkat E6.1 cells. The FRET level was 2.2 ± 0.6% (N = 17). A positive FRET sample in similar cells used the targeting of the Donor fluorophore (Alexa555) on a secondary antibody to a primary antibody carrying the Acceptor fluorophore (Alexa647) and targeting the TCR (CD3ε). The average FRET level of this positive control sample was 19.7 ± 0.8% (N = 13). Thus, our FRET imaging established a physical interaction of gp41(JRFL)ΔED* and TCR at the PM of T cells.

### Gp41 is enriched at the centre of the immune synapse (IS) upon TCR activation

**Figure 2:**
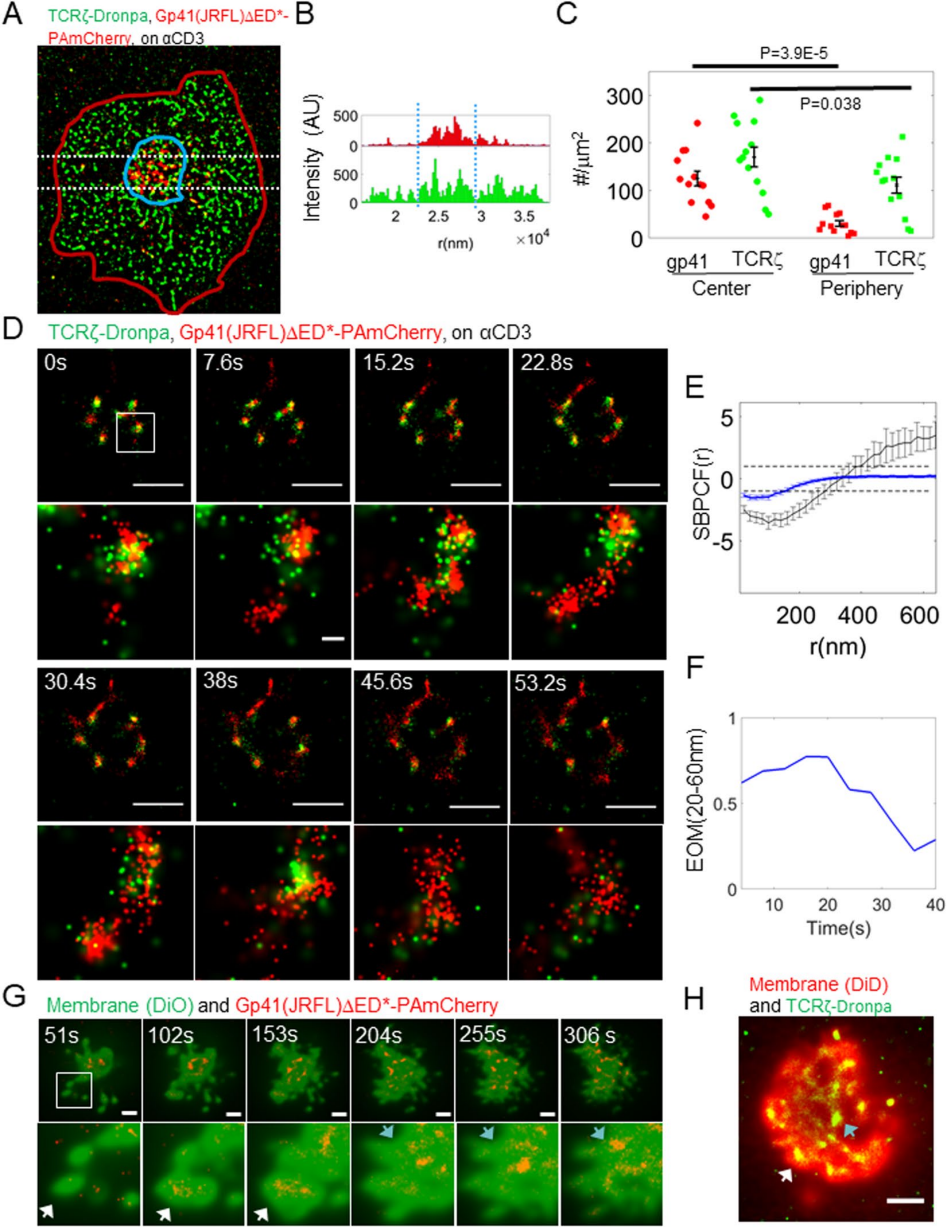
Gp41 and TCR are recruited together to the centre of the IS through early forming contacts and separate with cell spreading. (**A**) Gp41(JRFL)ΔED*-PAmCherry-enriched region at the centre (blue line) of the activated cells (red line) was manually identified. (**A**) Representative cell is shown (N = 12). (**B**) The distribution of molecular content of the shown cell in a horizontal cross section (white dotted lines in A). Blue dotted lines mark the horizontal edges of the central region in A. (**C**) The molecular content of these central regions was then compared to the surrounding areas (see also Table S1). (**D**) Two-colour PALM imaging of a live Jurkat E6.1 cell expressing TCRζ-Dronpa and gp41(JRFL)ΔED*-PAmCherry on an αCD3-coated coverslips. Each of the images was collected from 100 frames at a frame rate of 13.1 fps, yielding an effective frame time of 7.6 s. Bars – 2 μm. (**E**) Standardized bivariate PCF of TCRζ-Dronpa and gp41-PAmCherry (blue), compared to the 95% confidence interval of a Random labelling model (RL; black dotted lines at 1 and −1) and a model of no interaction (NI; black solid line). The correlation was averaged over the images in D for the shown clusters in the zoom and for all time-points. (**F**) The extent of mixing of TCRζ-Dronpa and gp41-PAmCherry, calculated for each wide area image in D. Error bars are SEM. (**G**) Two-colour TIRF imaging of a live Jurkat E6.1 cell expressing gp41(JRFL)ΔED*-PAmCherry (imaged via PALM) and the membrane stain DiO on an αCD3-coated coverslip. Each of the images was collected from 500 frames at a frame rate of 9.8 fps, yielding an effective frame time of 51 s. White arrowheads mark gp41 clusters in early segregated contacts; Cyan arrowheads mark gp41 clusters in flat and continuous membrane patches. A representative cell is shown (N = 9). Bars – 2 μm. (**H**) Two-colour TIRF imaging of a live Jurkat E6.1 cell expressing TCRζ-Dronpa (imaged via PALM) and the membrane stain DiD on an αCD3-coated coverslip. White arrowheads mark TCR clusters in early segregated contacts; Cyan arrowheads mark TCR clusters in flat and continuous membrane patches. A representative cell is shown (N = 9). Bar – 2 μm.

Strikingly, in our two-colour PALM images we note a morphological change to the recruitment of gp41 in the cell footprint upon TCR stimulation. While gp41(JRFL)ΔED* clusters were spread rather evenly across the cell footprint in non-activated cells (Fig. 1A), they preferably localized to the centre of the cells footprint in stimulated cells (Fig. 2A,B; observed also in Fig. 1D). To better quantify differences in gp41(JRFL)ΔED* recruitment between stimulated and unstimulated cells, we manually identified the gp41(JRFL)ΔED* enriched regions of the cell footprint and compared protein concentrations, areas, etc. between the inside and outside of these regions (see Fig. 2C and Table S1 for the complete comparison). Indeed, we found that gp41(JRFL)ΔED* was highly enriched at the centre of cell footprints of stimulated cells (p = ~4E-5). A significant, yet less pronounced, enrichment was also detected for the TCR (p = 0.038)^28^.

The correlated enrichment of gp41(JRFL)ΔED* and of the TCR at the centre of the cell footprint of stimulated cells raised the question about the mechanism that could form such a pattern. It is possible that gp41 arrives with the TCR at the PM using the same transport pathway or that it is nucleated in clusters by the TCR at the PM. To better resolve the patterning mechanism, we turned to two-colour PALM imaging of live cells, as they approach and spread on TCR-stimulating coverslips (Fig. 2D and Movies M1, M2). Our imaging showed that clustering of both TCRζ and gp41(JRFL)ΔED* occurred very early upon cell engagement of the coverslip, stimulation and spreading. Importantly, the two proteins closely associated in these clusters, as confirmed by the EOM and SBPCF curves (Fig. 2E,F). In fact, the SBPCFs were very close to the RL model Fig. 2E). This association reduced with cell spreading, as gp41(JRFL)ΔED* clusters and TCRζ became separated (Fig. 2D). Indeed, the EOM calculated for multiple individual clusters of these two proteins dropped over time (Fig. 2F). This effect was robustly detected in spite of the fast fading of the green emission of Dronpa, as newly detected TCRζ-Dronpa molecules mixed less with gp41(JRFL)ΔED* clusters (compare movies M2 and M1, with and without Kalman filtering for compensation of this effect; Analyses in Fig. 2E,F were conducted without Kalman filtering). Importantly, this time dependent separation explains the partial mixing of gp41(JRFL)ΔED* and TCR we detected earlier in fixed cells, as relatively newer and older protein clusters are imaged together and cannot be differentiated. Thus, their molecular interactions are averaged in the PALM images and in the EOM and SBPCF statistics. Note that on non-stimulating coverslips no such pattern exists and the dynamic adhesion of cells to the coverslip is hard to follow using our imaging technique. In our experiments, we were able to follow the cell footprint with a time resolution of 3.8 sec (Fig. 2D and Movies M1, M2).

Recent studies have identified membrane ruffling, and especially microvilli, as a mechanism for molecular clustering at early cell contacts with APCs^29^ and engaged surfaces^30,31^. To resolve this effect on gp41 and TCR clustering, we visualized live cells, expressing either gp41(JRFL)ΔED* or TCRζ, and general membrane stains in TIRF mode (Fig. 2G,H). We observe that gp41 is indeed included in early cell contacts (Fig. 2G; see zoom images at 50–150 sec; white arrowheads). With cell spreading on the coverslips, the membrane flattens and includes the gp41 clusters, but no further structure is evident. Similar patterns are also captured for TCR (Fig. 2H)^29–31^.

We conclude that membrane structure contributes to the recruitment of gp41 and TCR clusters into early cell contacts. Still, this effect does not account for the prolonged clustering and time-dependent colocalization patterns of these molecules (Figs 1 and 2B–F).

### Gp41-TCR interaction is not mediated by gp41 interactions with cholesterol

We next aimed to find the gp41 part that mediates gp41 interaction with the TCR, and hence focused on specific gp41 domains, as described below. The gp41 CRAC domain, a 5 aa motif (LWYIK), which is adjacent to the transmembrane domain of the protein has been shown to specifically bind cholesterol (PMID: 12488049). Recently, the CRAC motif has been suggested to promote gp41 association and self-clustering in cholesterol-rich lipid rafts^32^ via its affinity to cholesterol^19^. The TCR has also been suggested to depend on lipid-rafts for TCR stimulation^33,34^. Although this dependency remains controversial^35^, we tested whether gp41-cholesterol binding may facilitate the gp41-TCR interaction we have found. For this experiment, we introduced a mutation in the CRAC domain that abrogated gp41 association with lipid-rafts^19^ and potentially the interactions with the TCR if this process is based on a cholesterol dependent mechanism. The gp41(JRFL)ΔED*-mCRAC mutant was labelled with either PAmCherry or Dronpa (see Fig. S1B) and imaged via two-colour PALM with either gp41(JRFL)ΔED*, TCRζ or GPI, a well-known lipid raft marker^36^.

**Figure 3:**
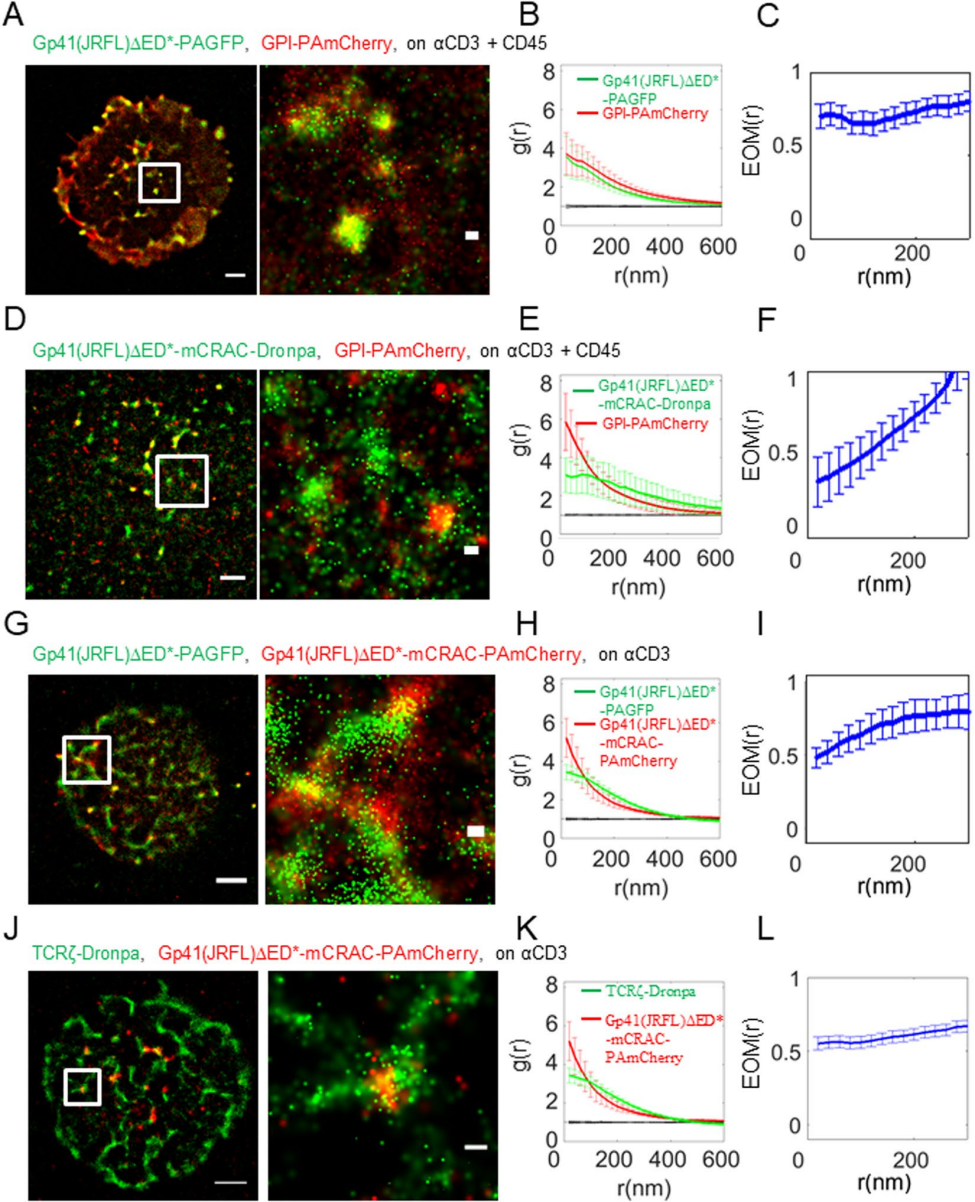
Gp41-TCR interaction is not mediated by gp41 interactions with cholesterol. (**A**) Two-colour PALM imaging of fixed E6.1 Jurkat cells expressing gp41(JRFL)ΔED*-PAGFP and GPI-PAmCherry on coverslips coated with an αCD3 and αCD45. Cells were dropped and let spread on the coverslip for 3 min before fixation. Bars – 2 μm (left) and 200 nm (right). Shown is a representative cell (N = 16). (**B**) PCF of gp41-PAGFP (green) and GPI-PAmCherry (red). (**C**) EOM of gp41(JRFL)ΔED*-PAGFP and GPI-PAmCherry. (**D**) Two-colour PALM imaging of fixed E6.1 Jurkat cells expressing GPI-PAmCherry and gp41-mCRAC-Dronpa on coverslips coated with an αCD3 and αCD45. Cells were dropped and let spread on the coverslip for 3 min before fixation. (**E**) PCF of gp41(JRFL)ΔED*-mCRAC-Dronpa (green) and GPI-PAmCherry (red). (N = 6). (**F**) EOM of gp41(JRFL)ΔED*-mCRAC-Dronpa and GPI-PAmCherry. (**G**) Two-colour PALM imaging of fixed E6.1 Jurkat cells expressing gp41(JRFL)ΔED*-PAGFP and Gp41(JRFL)ΔED*-mCRAC-PAmCherry on an αCD3-coated coverslips. Cells were dropped and let spread on the coverslip for 3 min before fixation. (**H**) PCF of Gp41(JRFL)ΔED*-PAGFP (green) and gp41(JRFL)ΔED*-mCRAC-PAmCherry(red). (N = 7). (**I**) EOM of gp41(JRFL)ΔED*-PAGFP and gp41(JRFL)ΔED*-mCRAC-PAmCherry. (**J**) Two-colour PALM imaging of fixed E6.1 Jurkat cells expressing TCRζ-Dronpa and gp41(JRFL)ΔED*-mCRAC-PAmCherry on an αCD3-coated coverslips. Cells were dropped and let spread on the coverslip for 3 min before fixation. (**K**) PCF of TCRζ-Dronpa (green) and gp41(JRFL)ΔED*-mCRAC-PAmCherry(red). (N = 16). (**L**) EOM of TCRζ-Dronpa and gp41(JRFL)ΔED*-mCRAC-PAmCherry. Error bars are SEM.

We started by confirming that gp41(JRFL)ΔED* and GPI closely associate at the PM of activated T cells (Fig. 3A–C; note that we used here coverslips coated with both αCD3 and αCD45 for improved cell adhesion). As expected, the mCRAC mutation significantly diminished this interaction, however did not abrogate it completely (Fig. 3D–F). This can be seen by the higher EOM value at the shortest length-scales of 0.7–0.8 and the closer proximity of the SBPCF curve to the RL model (relative to the NI model) for gp41(JRFL)ΔED* (Figs 3C and S2D) than for the mCRAC mutant (EOM of ~0.4 at 20 nm in Figs 3F and S2E). Accordingly, gp41(JRFL)ΔED* and the mCRAC mutant only partially colocalized at the PM (Fig. 3G–I) and did not completely mix (Figs 3I and S2F). Still, the interaction of gp41 and TCRζ was not affected much by the CRAC mutation. We found similar self-clustering of both gp41(JRFL)ΔED*-mCRAC and TCRζ as for the non-mutated gp41(JRFL)ΔED*, enrichment at the centre of the IS and co-clustering between these proteins (compare Figs 3J–L, S2G with Figs 1D–F, S2C). Since we could not find a detectable effect of the mCRAC mutation on either gp41(JRFL)ΔED* localization at the cell footprint or its association with the TCR, we conclude that gp41-TCR interaction is not mediated by gp41 interactions with cholesterol.

### Gp41-TCR interaction depends on the viral clone

Various HIV-1 clones have been isolated and may exhibit different characteristics, including their interactions with host proteins. Among these isolates, the JRFL and HXB2 stand out as they are widely used for HIV-1 research. HXB2 is a standard, lab-adapted, CXCR4-tropic HIV strain that is often used in basic HIV research. On the other hand, JRFL is a CCR5-tropic clinical isolate and therefore likely bears more physiological relevance. The similarity between the gp41 proteins derived from these isolates is high, but with some differences (see sequence alignment in Fig. S1C for Q75760 = JRFL and P04578 = HXB2).

**Figure 4:**
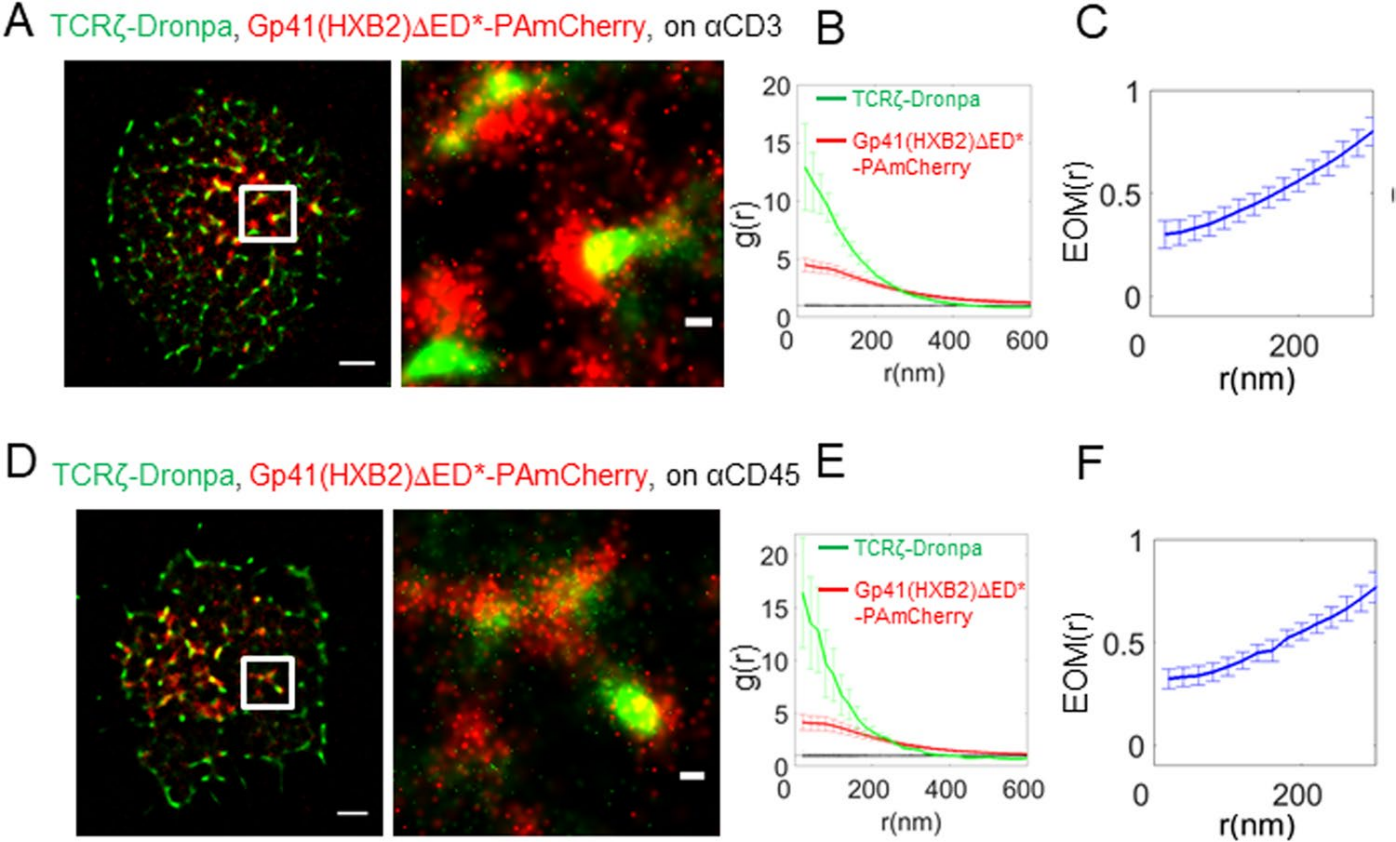
Gp41-TCR interaction depends on the viral clone. (**A**) Two-colour PALM imaging of fixed E6.1 Jurkat cells expressing TCRζ-Dronpa and gp41(HXB2)-PAmCherry on an αCD3-coated coverslips, where gp41 was derived from the HIV-1 HXB2 clone. Cells were dropped and let spread on the coverslip for 3 min before fixation. Bars – 2 μm (left) and 200 nm (right). Shown is a representative cell (N = 16). (**B**) PCF of TCRζ-Dronpa (green) and gp41-PAmCherry (red). (**C**) EOM of TCRζ-Dronpa and gp41(HXB2)-PAmCherry. (**D**) Two-colour PALM imaging of fixed E6.1 Jurkat cells expressing TCRζ-Dronpa and gp41(HXB2)-PAmCherry on an αCD45-coated coverslips. Cells were dropped and let spread on the coverslip for 3 min before fixation. Bars – 2 μm (left) and 200 nm (right). (N = 11). (**E**) PCF of TCRζ-Dronpa (green) and gp41(HXB2)-PAmCherry (red). (**F**) EOM of TCRζ-Dronpa and gp41(HXB2)-PAmCherry. Error bars are SEM.

We started our study with gp41 derived from the JRFL isolate (Figs 1–3). We next checked whether the isolate type could affect the interaction of gp41 with the TCR. For that, we repeated the imaging of T cells expressing TCRζ-Dronpa and HXB2 derived gp41-PAmCherry (gp41(HXB2)-PAmCherry; see Fig. S1B) using two-colour PALM. We found that these proteins significantly interact under either TCR stimulating or non-stimulating conditions (Fig. 4A,D). However, surprisingly, the TCR was more self-clustered in these cells, as indicated by the univariate PCF statistics (Fig. 4B,E) and the interaction between the proteins was weaker by the EOM and SBPCF statistics (Figs 4C,F and S2I,J; compare with Figs 1C,F and S2B,C for the related results of the JRFL construct). We have previously shown that co-clustering between two molecular species, as presented by bivariate PCF statistics, are largely independent of molecular densities of the interacting specifies [up to a factor of ~3 in the densities^13^].

### Gp41-TCR interaction is abrogated by its ectodomain in tight contacts within the IS and by mutation of the gp41 transmembrane domain

Next, we were interested in the molecular mechanisms that mediate the interaction of gp41 with the TCR. Although the gp41 TMD seems to be the crucial factor of gp41-TCR interactions^37^, other parts of the gp41 ED might be involved as well^5,6^. To specifically address this question, we prepared suited variants of our HXB2 derived protein. New constructs were generated by addition of the gp41 ED as well as a variant of this protein in which the TMD was replaced with the TMD of CD8 (Fig. S1B, marked as gp41(HXB2) and gp41(HXB2)-mTMD).

**Figure 5:**
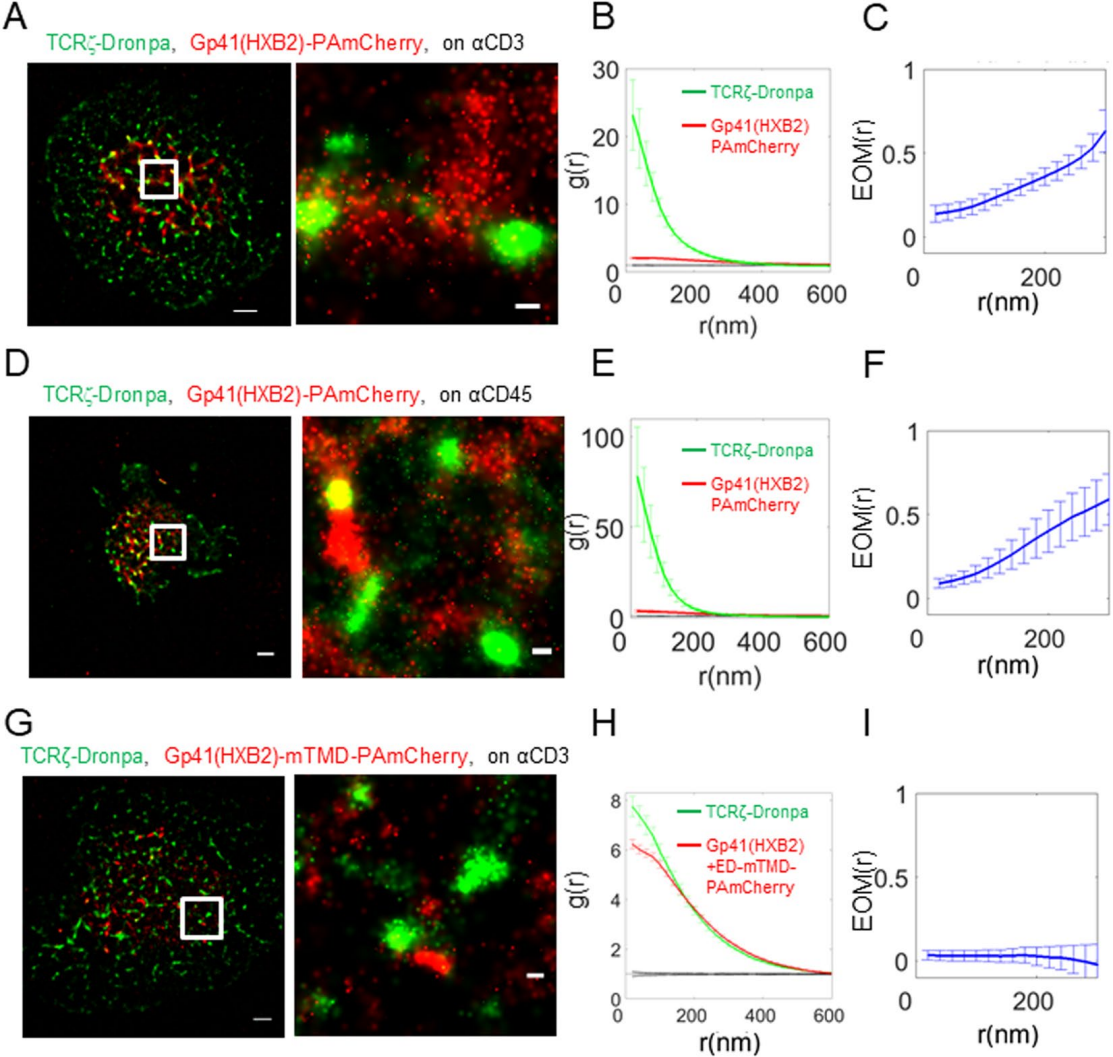
Gp41-TCR interaction is abrogated by its ectodomain in tight contacts within the IS and by mutation of the gp41 transmembrane domain. (**A**) Two-colour PALM imaging of fixed E6.1 Jurkat cells expressing TCRζ-Dronpa and gp41(HXB2)-PAmCherry on an αCD3-coated coverslips. Cells were dropped and let spread on the coverslip for 3 min before fixation. Bars – 2 μm (left) and 200 nm (right). Shown is a representative cell (N = 24). (**B**) PCF of TCRζ-Dronpa (green) and gp41(HXB2)-PAmCherry (red). (**C**) EOM of TCRζ-Dronpa and gp41(HXB2)-PAmCherry. (**D**) Two-colour PALM imaging of fixed E6.1 Jurkat cells expressing TCRζ-Dronpa and gp41(HXB2)-PAmCherry on an αCD45-coated coverslips. Cells were dropped and let spread on the coverslip for 3 min before fixation. Bars – 2 μm (left) and 200 nm (right). (N = 8). (**E**) PCF of TCRζ-Dronpa (green) and gp41(HXB2)-PAmCherry (red). (**F**) EOM of TCRζ-Dronpa and Gp41(HXB2)-PAmCherry. (**G**) Two-colour PALM imaging of fixed E6.1 Jurkat cells expressing TCRζ-Dronpa and gp41(HXB2)-mTMD-PAmCherry on a TCR stimulating, αCD3-coated coverslips. Cells were dropped and let spread on the coverslip for 3 min before fixation. (N = 25). (**H**) PCF of TCRζ-Dronpa (green) and gp41(HXB2)-mTMD-PAmCherry (red). (**I**) EOM of TCRζ-Dronpa and gp41(HXB2)-mTMD-PAmCherry. Error bars are SEM.

The ED of gp41 has a 9 nm rod-like structure^38^, which is comparable to the 7 nm extracellular extension of the TCR and the ~13 nm intermembrane distance induced by TCR-pMHC interaction^39^. Thus, we hypothesized that the bulky ED of gp41 in dense gp41 clusters could interfere with TCR-pMHC interactions in tight contacts within the IS^25,40^. To study the putative role of the ED in gp41-TCR interactions, we used a construct derived from HXB2 that included the entire ED and imaged it, upon co-expression with TCRζ using two-colour PALM (Gp41(HXB2), shown in Fig. S1B). We found that these two proteins showed clusters that closely localized, but did not overlap (Fig. 5A), yielding EOM and SBPCF statistics that approached the model of no interaction (Figs 5C and S2K). Interestingly, TCRζ seemed to self-cluster to a much higher extent relative to the self-clustering extent of the gp41(HXB2)ΔED* construct (compare Fig. 5B,E to Fig. 4B,E, and to Fig. 1B,E). The interaction between gp41(HXB2)ΔED* and TCRζ was not dependent on TCR activation, as it occurred also on coverslips coated with αCD45 (Figs 5D,F and S2J). We further used the gp41(HXB2) construct to study the cellular localization of this protein (Fig. S4). Two-colour confocal imaging (Fig. S4A) of this construct and TCRζ-Dronpa in fixed cells showed the expected presence of TCRζ at the PM of cells, while the majority of the gp41(HXB2) proteins resided in intracellular membranes, most likely in the ER. Since it is hard to distinguish the ER from the PM in T cells due to their small cytosolic volume and the insufficient resolution of diffraction limited microscopy, we turned to imaging these proteins using two-colour PALM of fixed cells at cross sections at and above the coverslip. Indeed, this imaging (Fig. S4B) better resolved the PM and the ER, and showed that although these proteins significantly interacted at the PM (top and bottom rows; filled white arrows), most of the proteins existed in separate pools (empty white arrows), as found by confocal imaging (Fig. S4A). The distinct enrichment of gp41 at the ER and of the TCR at the PM suggests that a small pool of gp41 and TCR may interact outside of the PM and traffic together to the PM; Alternatively, these molecules may get enriched in early cell contacts (Fig. 2G,H), esp. at the tips of engaged microvilli^30,31^, by their recruitment from other parts of the PM.

Importantly, the gp41 TMD has been shown before to be responsible for gp41-TCR interactions^5^. Specifically, the Shai group has shown that peptides encoding the TMD of gp41 closely associate with TCR through a GxxG motif^5^. To test the role of the TMD in our assay, we turned again to two-colour PALM imaging of gp41(HXB2)-mTMD and TCRζ. We found that this construct completely abrogated the interaction of gp41 and TCR at the IS (Fig. 5G–I). Importantly, the EOM and SBPCF curves of these two proteins followed the model of no-interaction (Figs 5I and S2L), while the self-clustering of gp41(HXB2) was not much affected by the expression of the TMD mutant (Fig. 5H), and was similar to the gp41 constructs without the ED (Figs 1E and 4B). Since the TMD mutants lay farther from TCR clusters than the full length gp41 (compare Fig. 5G and Figs 4A,D or 5A,D), we conclude that this separation allows for more uniform spreading of TCRs at the PM by a yet unknown mechanism. This change in TCR self-clustering may be further specific to differences between the JRFL and HXB2 isolates, as most HXB2 constructs (with the exception of the TMD mutant) showed enhanced self-clustering of the TCR in comparison to the JRFL constructs.

To conclude our results using the different gp41 constructs, our imaging and statistics show the high interaction of the TCR with gp41 for the JRFL gp41 constructs without the ED (EOM of 0.5–0.7, panels 1C,F). Note that the EOM between the two proteins slightly increased under non-stimulating conditions (Fig. 1C), in comparison to stimulating conditions (Fig. 1F) while using the mCRAC mutant did not change this measure under activating conditions (Fig. 3J). Similar constructs without the ED derived from HXB2 showed reduced interactions (EOMs starting from ~0.3 ± 0.05 at the shortest length scales, Fig. 4C,F). This interaction was significantly diminished in the IS for the full-length gp41 constructs that included the ED (EOMs starting at ~0.1–0.15, Fig. 5C,F). A replacement of the gp41 TMD with a CD8 TMD in full length constructs resulted in the complete abrogation of the interaction (EOM of ~0.05, Fig. 5I). We thus established that gp41 closely interacts with the TCR regardless of cell activation, that gp41-TCR interaction is affected by the isolate type, that it is diminished by its ED in tight contacts within the IS and that this interaction is completely abrogated by an added mutation of the gp41 transmembrane domain.

### Gp41 promotes TCR phosphorylation on ITAMs

**Figure 6:**
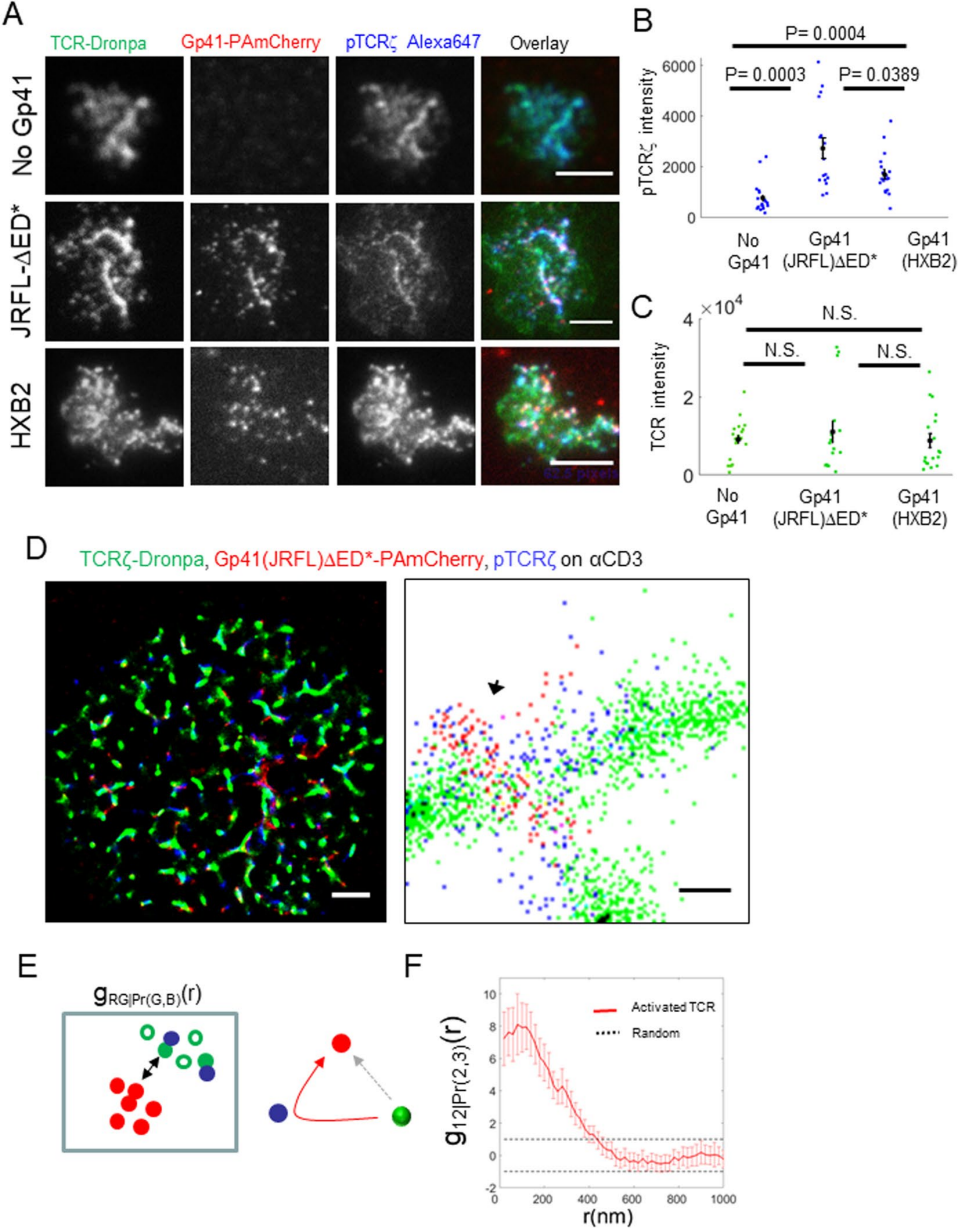
Gp41 promotes TCR phosphorylation and correlates with localized TCR activation. (**A**) Three-colour diffraction limited microscopy of fixed E6.1 Jurkat cells expressing either TCRζ-Dronpa alone or with gp41(JRFL)ΔED*-PAmCherry or gp41(HXB2)-PAmCherry and stained for pTCRζ on an αCD3-coated coverslips. Cells were dropped and let spread on the coverslip for 3 min before fixation. Bars – 2 μm (left) and 200 nm (right). Shown are representative cells (No gp41, N = 19; Gp41(JRFL)ΔED*, N = 16; gp41(HXB2), N = 18). (**B**) The total intensity of phosphoTCRζ (pTCRζ) detected at the footprint of individual cells in the samples shown in panel A. (**C**) The total intensity of pTCRζ detected at the footprint of individual cells in the samples shown in panel A. Mean values and SEMs are shown for each sample type in panels A and B. (**D**) Three-colour SMLM imaging of fixed E6.1 Jurkat cells expressing TCRζ-Dronpa and Gp41(JRFL)ΔED*-PAmCherry (via PALM) and stained and imaged for phosphoTCRζ (pTCRζ) (dSTORM) on an αCD3-coated coverslips. Cells were dropped and let spread on the coverslip for 3 min before fixation. Bars – 2 μm (left) and 200 nm (right). In zoom, arrows point to representative clusters of high colocalization between gp41(JRFL)ΔED*-PAmCherry and pTCRζ. Shown is a representative cell (N = 9). (**E**). (left) A scheme for the analysis of phosphorylation enhancement of TCRζ by gp41. First, we selected TCRζ (green) molecules by their proximity to pTCRζ (blue) molecules (selected molecules are marked with filled circles while unselected molecules are marked with empty circles). We then compared the bivariate PCF of these proximity-selected TCRζ (green) molecules with gp41 (red) molecules to the interaction of the same number of randomly selected TCRζ (green) molecules to the gp41 (red) molecules. (right) The inferred scheme of molecular interaction, according to the statistics on left. The red arrow indicates that gp41 correlates with (or promotes) enhanced phosphorylation of TCRζ (green, proximal to blue). The dotted gray arrow marks that TCRζ molecules have an equal probability of being phosphorylated, regardless of their interaction with gp41. (**F**) Conditional standardized BPCF (SBPCF) of gp41(JRFL)ΔED*-PAmCherry (red) with a TCRζ-Dronpa (green) subpopulation that was selected based on its proximity to phosphorylated TCRζ see main text and analyses section of the SI for further details).

Since we found that gp41 interacts with the TCR, we hypothesized that gp41 could affect early T cell activation. To study such a potential affect, we imaged using diffraction-limited microscopy cells that expressed either TCRζ-Dronpa alone, or with co-expression of Gp41(JRFL)ΔED*-PAmCherry or Gp41(HXB2)-PAmCherry. The cells were dropped on αCD3ε stimulating coverslips and stained for phosphoTCRζ on one of its immunoreceptor tyrosine-based activation motifs (ITAMs), using a primary antibody (pY83 of TCRζ) and a secondary antibody stained with Alexa647 (Fig. 6A). We next measured the total intensity of phosphoTCRζ and TCR in individual cells of the different samples. Strikingly, we found that gp41-expressing cells had 2.2 and 3.6 fold (for Gp41(HXB2) and Gp41(JRFL)ΔED*, respectively) higher levels of phosphoTCRζ at their interface with the coverslips (Fig. 6B). This difference was not due to changes in the total number of available TCRs at the interface, as TCR levels were similar for all samples (Fig. 6C). Thus, we found that gp41 expression promotes phosphorylation of ITAMs of the TCRs and consequently, early T cell activation.

### Gp41 correlates with localized TCR activation

Next, we studied whether the enhancement we found in TCR phosphorylation in gp41-expressing cells was due to the physical interaction of gp41 and the TCR that we have found. Since the cell contains TCRs that are clearly not associating with gp41, we compared the extent of TCR phosphorylation of TCRs proximal to gp41 with the extent of phosphorylation of the overall population of TCRs. This assay required the development of multiple techniques, as follows. First, we needed to visualize gp41, TCR and phosphorylated TCRs in the same cells and in single molecule detail. For that, we conducted three-colour SMLM of these constructs (Fig. 6D), as follows. Gp41(JRFL)ΔED*-PAmCherry and TCRζ-Dronpa were imaged by two-colour PALM. The cells were also stained for phosphoTCRζ using a primary antibody (pY83 of TCRζ) and a secondary antibody stained with Alexa647, such that phosphorylated TCRζ chains could be imaged by direct Stochastic Optical Reconstruction Microscopy (dSTORM)^41^ in the same cells. Second, we needed to analyse the resultant images, looking for the effect of gp41 proximity to TCR on TCR phosphorylation. Here, we turned to conditional SBPCF statistics that we have recently introduced^42^ (see Fig. 6E and Analyses section in the SI for further details). Briefly, TCRζ proteins were selected based on their proximity (<36 nm) to phosphorylated TCRζ proteins (see SI Analyses section in the SI for further details). The SBPCF of the interaction between this subpopulation of TCRs and gp41 was compared to the SBPCF of the same number of randomly selected TCRs to gp41, as a null hypothesis. We found that the conditional SBPCF curve exceeded the 95% confidence interval of the null hypothesis (Fig. 6F). This shows that the gp41-TCR interaction correlates with phosphorylated TCRs, while rejecting the alternative hypotheses that gp41 does not correlate or negatively correlates with TCR phosphorylation. In contrast, cells on αCD45- or αCD11-coated coverslips showed much-reduced (yet, non-zero) levels of pTCRζ staining (Fig. S7A). Under these conditions we also found a correlation of TCR phosphorylation and gp41 (Fig. S7C,E), similar to our findings for cells on αCD3-coated coverslips (Fig. 6D–F). Our results suggest that residual TCR activation may be promoted by gp41. Still, residual activation of the cells by the αCD11- and αCD45-coated coverslips cannot be ruled out.

### Gp41 enhances CD69 upregulation followed by massive cell death

**Figure 7:**
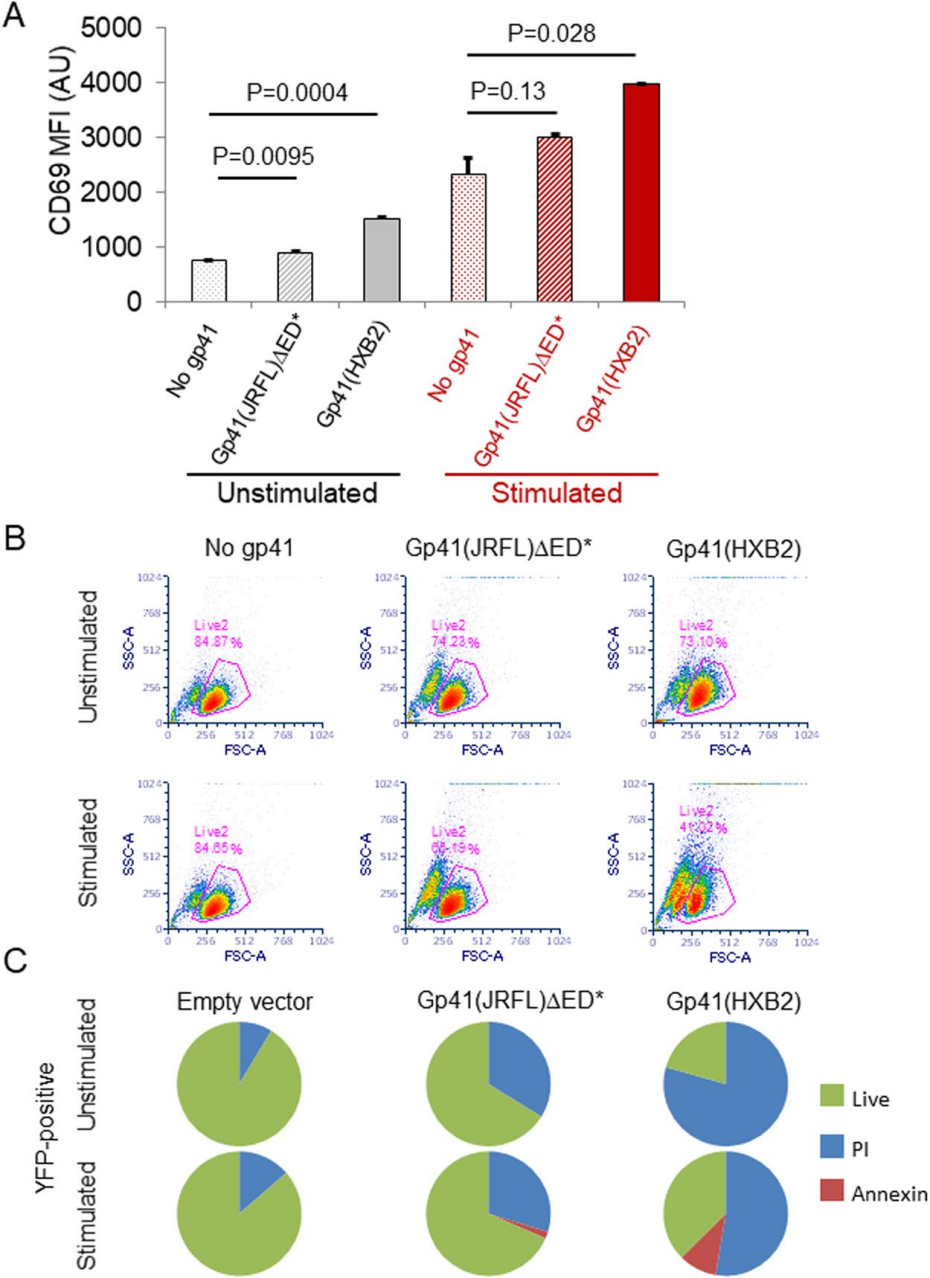
Gp41 leads to massive cell death via necrosis and promotes CD69 upregulation. (**A**) Mean fluorescence intensity (MFI) of CD69 upregulation by Jurkat E6.1 cells, expressing gp41 constructs (either gp41(JRFL)ΔED*-YFP or gp41(HXB2)-YFP) or YFP alone (‘no gp41’), with or without TCR stimulation. CD69 was stained using Alexa647. Data were collected using flow cytometry, and included gating for live cells and for positive YFP expression. The average MFI and error-bars (SEM) were calculated from triplicate experiments. (**B**) Flow cytometry data of forward and side scattering by Jurkat E6.1 cells, expressing gp41 constructs (either gp41(JRFL)ΔED*-YFP or gp41(HXB2)-YFP), with or without TCR stimulation. Magenta lines and numbers indicate the gate and fraction of live cells. (**C**) Death analysis of Jurkat E6.1 cells, expressing (by gating) either YFP, gp41(JRFL)ΔED*-YFP or gp41(HXB2)-YFP, with or without TCR stimulation. Cells were stained using necrosis (PI) and apoptosis (Annexin) markers and analysed using flow cytometry 24 hrs post transfection.

Next, we were interested in the effect of gp41 on downstream markers of T cell activation, namely the upregulation of CD69. For that, we transfected Jurkat E6.1 cells with either gp41(JRFL)ΔED*-YFP, gp41(HXB2)-YFP or with YFP alone. Transfected cells were incubated for 24 hrs, and then were either stimulated using 0.5 μgr/ml αCD3ε in suspension for 3 hrs, or kept for that time under non-stimulating conditions. Next, cells were stained for surface CD69 with Alexa647 (see Material and Methods in the SI for further details). Gating on YFP-positive cells after 24 hrs, we observed that gp41 expression (esp. of the HXB2 isolate) also resulted in higher levels of CD69 upregulation, both in unstimulated and in stimulated cells (Fig. 7A). We found similar results by diffraction-limited imaging of cells on antibody-coated coverslips (Fig. S6B,D).

Surprisingly, using flow-cytometry and imaging, we observed significantly smaller fractions of live cells according to FSC-SSC gating for cells that expressed either one of the gp41 constructs (Figs 7B and S6A,C and zoom bright-filed images in Fig. S6D). Interestingly, unstimulated cells expressing Gp41(HXB2)-YFP further underwent massive death after 48 hrs, showing a live-cell count of 25 ± 0.5% in comparison to the cells without gp41. At that time, cells expressing gp41(JRFL)ΔED*-YFP showed a much lower drop in live-cell count, to 85.4 ± 2%.

Next, we studied the mechanism of the induced cell death by transfecting cells with either YFP, Gp41(JRFL)ΔED*-YFP or Gp41(HXB2)-YFP, and staining them with markers for apopotosis (APC-Annexin V) and necrosis (PI). Gating again on YFP-positive cells after 24 hrs, we assessed necrosis as the dominant mechanism for cell death under both stimulating and non-stimulating conditions (Fig. 7C). It is possible that the PI-stained fraction of cells presented in Fig. 7C indicates cells that are not completely dead, but rather have a compromised PM that allows PI entry and cell staining.

Thus, we conclude that gp41 can lead to CD69 upregulation in unstimulated cells and to enhanced CD69 upregulation in stimulated cells. Interestingly, we note that the HXB2 construct was more potent than the JRFL construct in CD69 upregulation (Figs 7A, S6B,D) and in inducing cell death (Figs 7B,C and S6A,C).

## Discussion

Here, we applied SMLM in two and three-colours to study the assembly of gp41 at the PM of fixed and live CD4^+^ T cells. Second order statistical analyses allowed us to detect significant interactions of gp41 with the TCR in activated and non-activated T cells. FRET imaging confirmed the physical interaction of the two proteins at the PM of the cells, in accord with the SMLM results, and previous biochemical and biophysical results using gp41 derived peptides^5,6^. The interaction between the TCR and gp41 seems to be not well structured and deterministic, since both molecules show heterogeneity in their extent of co-clustering between different clusters across the PM. Importantly, we found that gp41 was enriched at the attachment sites of cells activated with antibody-coated coverslips. Live cell PALM imaging showed that this enrichment was due to a dynamic process, namely the simultaneous recruitment of gp41 and TCR at early contacts of the cells. With time, the two proteins separated such that gp41 remained at the centre of the cell contact. With further cell spreading, new TCRs, but not many gp41 molecules, appear at newly forming areas at the periphery of the cell footprint, thus showing the concentric pattern as in Fig. 2A.

Our assay of imaging cells on antibody-coated coverslips has been used in multiple previous studies to mimic key aspects of the IS, including cell spreading, TCR-specific stimulation, molecular clustering, and signalling downstream the TCR^43,44^. Note that this assay restricts the physiological mobility of the membrane proteins that are targeted by the antibodies on the coverslips (e.g. TCR targeting by αCD3ε). Such mobility is better captured by cell imaging on glass-supported lipid bilayers (e.g.^28^). Still, our assay has enabled the detection of the gp41 interaction with the TCR in live, interacting cells.

We further note that in our assay, overcounting of molecules and membrane ruffling could contribute to apparent clustering of both gp41 and TCR. While our SMLM analyses were aimed at minimizing molecular overcounting (see Methods), the contribution of this effect on molecular self-clustering cannot be completely ruled out. Furthermore, our imaging indicates involvement of membrane ruffling in TCR and gp41 clustering in early cell contacts (Figs 1 and 2B–F) but not in the prolonged clustering and colocalization of these molecules (Fig. 2G,H).

We next turned to resolve the mechanism of the gp41-TCR interaction using mutants and truncation constructs of gp41. First, a CRAC mutant of gp41, showing a reduced colocalization with the raft marker protein GPI-PAmCherry exhibits similar interaction with TCR as wt gp41, thus excluding the involvement of cholesterol dependent raft partitioning. In contrast, the extent and spatial organization of the gp41-TCR interaction significantly differed between ED-deficient and full-length constructs [gp41(HXB2)ΔED* vs. gp41(HXB2)]. Gp41(HXB2)ΔED strongly mixes with TCR clusters, while gp41(HXB2) localized to the periphery of TCR clusters. Furthermore, a gp41 mutant in which the TMD was replaced with the TMD of CD8 [gp41(HXB2)-mTMD] was not able to interact with the TCR. Thus, the gp41 TMD is responsible for gp41-TCR interactions, as previously shown using gp41 derived peptides^5,6^. Interestingly, the TMD mutant still showed self-clustering, indicating differential mechanisms of gp41 self-clustering and its association with the TCR.

Altogether, we showed that gp41 closely interacts with the TCR regardless of cell activation, and that this interaction is affected by the tropism/virus isolate, being more pronounced for the CCR5-tropic JRFL than for the lab-adapted, CXCR4-tropic HXB2. Still, we could not rule out differences in the results between these isolates due to the missing ED for the JRFL isolate vs. the full length of the HXB2 isolate. We further showed that this interaction is significantly reduced by the gp41 ED in tight contacts within the IS and that this interaction is completely abrogated by an added mutation of the gp41 TMD.

Based on our results, we propose that gp41 and the TCR closely associate at the PM of non-activated CD4^+^ T cells and that this interaction is mediated by the gp41 TMD region. Upon cell adhesion and spreading, the bulky ED of gp41 leads to its segregation from the TCR in the tight contact. This could explain the strikingly high self-clustering of the TCR that we found at the PM of cells that co-expressed gp41(HXB2) and spread on coverslips in our imaging assay. A similar mechanism has been suggested for the kinetic segregation of the TCR from bulky glycoproteins such as CD45^45,46^. Since CD45 acts as a phosphatase, its segregation from TCRs in the IS is suggested to allow for their phosphorylation and signalling downstream.

Our study focuses on the interaction of gp41 with cellular the TCR. Interestingly, both gp41 self-clustering and its co-clustering with the TCR did not require other HIV-1 proteins. Still, we note that other cellular and viral proteins (such as gag and gp120, which are missing in our assays) could significantly affect these molecular interactions under physiological conditions^3^. Thus, our study sets the stage for the future study of gp41 assembly and its interaction with the TCR in HIV-1-infected cells.

After finding the close interaction between gp41 and the TCR and its characterization, we were interested in the functional role of this interaction and its effect on T cell activation. We first hypothesized that the association of gp41 and the TCR could facilitate gp41 self-clustering, since gp41 could use TCR clusters as nucleation sites for self-clustering and viral assembly. However, the ability of the gp41 TMD mutant to self-cluster independent of TCR interaction argues against this idea. A second possible consequence of gp41 interaction with the TCR is that gp41 could directly affect the efficiency of TCR activation. Using FRET assays, the Shai group has shown that gp41 derived peptides encoding its TMD could reduce long-term T cell activation (upregulation and cytokine secretion) via close interactions with chains of the TCR complex^5,6^. We and others found an opposite effect of gp41 on T cell activation. Postler and Dorsier found that the cytoplasmic domain of gp41 activates cells via the NFKB pathway^47^. Here, we show that gp41 expression (from either the JRFL or HXB2 isolates) and its presence at the PM of activated T cells enhanced the levels of phosphoTCR at the interface, and thus, early T cell activation. Using three-colour SMLM and conditional second order statistics, we found that gp41 localization at the cell interface correlated preferentially with phosphorylated TCRs. That is, we found that gp41-TCR association colocalized with phosphorylated TCRζ, while not affecting the TCR ability to get phosphorylated and activated. Thus, we conclude that the gp41-TCR interaction accounts for the enhancement of TCR activation we observed in gp41-expressing cells. Using flow-cytometry and imaging, we further found that gp41 expression, esp. of the HXB2 construct, resulted in enhanced CD69 upregulation in either unstimulated or stimulated T cells.

These results seem to contrast the previously found deleterious effect of gp41-derived peptides on TCR activation. This contradiction could be a result of the varying experimental model systems, activation stimuli and activation readouts applied in the different studies. Moreover, it is conceivable that the various active parts of gp41 exert different immunomodulatory functions, depending on the cellular context, individual protein conformation or the overall gp41 expression level. Still, it can also not be excluded that immunosuppressive activities are specific to peptides and do not entirely reflect the mechanisms, induced by expressed proteins. To conclude, we showed that gp41 promotes TCR activation via its direct interaction with the TCR in early contacts within the IS, which are also sites of localized TCR activation^25,48^. Gp41 expression also resulted in enhanced T cell activation after 4 hours, and in massive cell death via necrosis after 24–48 hrs. These effects could significantly affect the viral life-cycle within host CD4^+^ T cells and their efficacy in mounting an immune response.

Taken together, our results shed new light on the dynamic process of gp41 assembly at the PM of host CD4^+^ T cells, and its relation to T cell activation via the TCR. Our methods are relevant to the study of arbitrary protein complexes in single molecule detail, and our results revealed a new mechanism of viral interaction with T cell signalling and activation.

## Materials and Methods

We briefly describe the materials and methods below, and provide further details in the Supplementary Materials and Methods.

### Samples

In this work gp41 was derived either from HXB2 or from JRFL (Fig. S1B,C). The JRFL derived proteins contained three LLP, TMD and MPER domains that were tagged by PAGFP and PAmCherry fluorophores. We constructed the single-point mutation from leucine (L) to isoleucine (I) to generate a mutation affects CRAC motif properties^19^, such as gp41 localization on cell membrane, which we studied here. We used EGFP-N1 or EGFP-C1 vectors (Clontech) to generate proteins tagged with the PAFPs PAmCherry, Dronpa (MBL International Corporation), and PAGFP. New constructs included wild-type gp41 and mutants, tagged with either Dronpa, PAmCherry or PAGFP. Constructs were validated by DNA sequencing. A Neon electroporation system (Invitrogen) was used to transfect Jurkat E6.1 T cells with DNA constructs, for expression of the fluorescently-tagged proteins. We checked transiently transfected cells for positive expression and conducted imaging within 48–72 hours from transfection. A stable cell line expressing TCRζ-Dronpa was maintained with Geneticin at 1.5 mg/ml (G418, Invitrogen) and was available for this study from previous published studies^25,42^. Cells evaluation and imaging included confocal microscopy, epifluorescence, TIRF, PALM and dSTORM imaging, as described below. Cell spreading and imaging was conducted on glass coverslips (#1.5 glass chambers, LabTek and Ibidi), as previously described^43^. Coverslips were coated with previously validated antibodies^23,44^: A purified mouse αhuman CD3ε (clone Ucht1; eBioscience 16–0038) served for direct stimulation of the TCR, causing robust T cell activation and spreading. An αCD45 antibody (BD Biosciences; 555480) was used for causing T cell spreading without directly triggering the TCR. Cleaned coverslips were incubated with either 10 μg/ml of stimulatory or non-stimulatory antibodies at 4 °C overnight or at 37 °C for 2 hours. Coverslips were next washed with PBS. Before imaging, cells were resuspended in imaging buffer (1 million cells per 150 μl). For imaging, we dropped 100,000–500,000 cells onto coverslip, incubated them at 37 °C for spreading (typically 3 min), and them fixed with 2.4% Paraformaldehyde (PFA) for 30 min at 37 °C. See SI for further details on reagents and sample preparation protocols.

### Imaging

We conducted two-colour PALM imaging similar to previous studies^23^, using a total internal reflection (TIRF) Nikon microscope with a CFI Apo TIRF X100 oil objective (NA 1.49, WD 0.12 mm). PAGFP, Dronpa and PAmCherry were photoactivated using variable intensity (0.5–10%) of 405 nm laser illumination and alternate excitation using (80–100%) 488 nm laser excitation (for PAGFP and Dronpa) and 561 nm for PAmCherry. Movies of fixed and live cell imaging were acquired for up to 3000 frames at 13.1 fps of an EMCCD Ixon^+^ camera. FRET imaging was conducted using the same Nikon microscope, with detection channels of 600 ± 25 nm (filter ET 600/50 M 265196) for the Donor (Alexa555) and 700 ± 37.5 nm (filter ET 700/75 M 263043) for the Acceptor (Alexa647). Confocal microscopy was conducted using an Olympus FV-1000 confocal microscope with a PLAPON60XO NA: 1.42, and similar excitation lasers for imaging PAGFP, Dronpa and PAmCherry. Three-colour SMLM was conducted using the same microscope by combining two-colour PALM of Gp41-PAmCherry and TCRζ-Dronpa with dSTORM imaging of phosphoTCRζ using a primary antibody (pY83 of TCRζ; Abcam) and a secondary antibody stained with Alexa647. These proteins were also imaged by diffraction-limited microscopy for quantifying the levels of proteins at the interface of the cells (see Fig. 6).

### Analyses

We used the ThunderSTORM software^49^ to analyse PALM and dSTORM movies and generate images. Briefly, this software served to identify individual peaks in the PALM and dSTORM movie frames, to correct for image drift, and to group peaks and assign them to individual molecules for rendering of the PALM images. A distance threshold and a temporal gap were employed for peak grouping to account for possible molecular blinking^12^. Temporal gaps were tested for each fluorophore separately to minimize possible overcounting of molecules. Individual molecules were presented in corresponding to the probability density values of their fitted Gaussian. This scale was set for each species separately, according to its maximal probability density in the field (note that it should not be interpreted as the density of molecules in clusters). See SI for further details on FRET analyses.

### Second order statistics

A Detailed description of the second order statistics used in this study is provided in the SI due to the length of these subsections. This description includes the univariate PCF, the EOM and SBPCF statistics and their relation to BPCF, and conditional second order statistics (Fig. 6E,F). We present these published statistics with a few changes, as explained briefly below (see definitions and further details in the Analyses part of the SI).

The BPCF detected is compared to nineteen simulated realizations of a ‘Random Labelling’ (RL) model, where the labels of the proteins are randomized while their detected positions are kept. If the two proteins homogeneously mix in the measured point patterns, the measured PCF would lie in the 95% confidence interval generated by the lowest and highest PCF values of the simulated realizations. Agreement of the data with the RL model indicates homogeneous mixing, and hence strong interaction (in a statistical sense) of the two species under study. Prior knowledge on the physical binding of the two species (e.g. from biochemical assays) can then help to interpret the studied interactions as physical binding events of the species. If the two proteins do not interact, the BPCF (g_12_) would yield a value of 1 [e.g. Fig. S2A (left); no-interaction (NI) model]. Still, the confidence interval due to the RL model is individual to each measured realization (i.e., to each cell). This prevents the averaging of the resultant BPCF statistics over multiple cells^13^. The EOM normalizes the BPCF curves and spans their range of possible values between the RL model (given a value of 1) and the NI model (given a value of 0) [e.g. Fig. S2A (right)]. This statistics is especially revealing at relatively short length-scales (0–300 nm) where the BPCFs of the RL and NI models separate well and where statistical interactions of molecules within the same cluster and between proximal clusters dominate. Thus, this measure becomes relative, intuitive and can be easily compared between multiple cells. However, this measure loses information about the shape of the BPCF curve. For that we introduce here a complementary measure, the SBPCF, which is constructed as follows. We standardized the BPCF first (see section on Analyses in the SI), thus allowing these statistics to be averaged over multiple cells. In this standardized form, the 95% confidence interval due to the RL model is limited by the values of +1 and −1, per definition. An SBPCF curve due to homogeneous mixing would fit in this confidence interval [e.g., dotted black lines in Fig. S2A (middle)]. Alternatively, the SBPCF of non-interacting species is taking negative values that are significantly smaller than −1 [NI model as a black line; e.g. Fig. S2A (middle) and Fig. 1C,F]. Typically, the measured SBPCF takes a negative value between these models. Variability of the SBPCF curves between multiple cells can now be expressed as error-bars (SEM) around the SBPCF curve and the NI model. Throughout this work we present both the EOM (in the main figures) and the SBPCF (typically in the SI) for clarity and completeness. We rely on these statistics in our conclusions, as they account for potential cell-to-cell variability. For the pair-correlation analyses, we chose for each cell a single wide region-of-interest in its visible footprint (typically covering most of the footprint), while excluding cell edges.

### Controls for SMLM imaging and analyses

To assess the effect of gp41 on TCR clustering, we visualized E6.1 cells expressing TCRζ alone (i.e. without gp41). Our imaging and PCF analyses recovered the significant self-clustering of TCRζ on both αCD3- and αCD45-coated coverslips and the significant increase in clustering extent (evident through the height of the PCF curve at the shortest scales) upon direct TCR stimulation, in accordance with previous studies^23–25^. We have further tested our two- and three-colour SMLM imaging by multiple control samples based on Jurkat E6.1 cells on TCR stimulating coverslips. First, we reversed the colours between gp41 and TCRζ, namely imaging Gp41(JRFL)ΔED*-PAGFP and TCRζ-PAmCherry (Fig. S5A). This imaging could capture the significant interaction between gp41 and TCRζ at the PM of the cells (Fig. S5B–D). We further tested our PALM imaging of gp41 using two different colours, namely imaging Gp41(JRFL)ΔED*-PAGFP and Gp41(JRFL)ΔED*-PAmCherry (Fig. S5E). Although the self-clustering of the two proteins was different between the two-colours due to different expression levels (Fig. S5F), the two proteins demonstrated high SBPCF (Fig. S5E) and EOM (Fig. S5H) that were, as expected, very close to the RL model. To validate the antibody staining of pTCRζ (pY83), we imaged cells stained with it via dSTORM, together with imaging TCRζ-Dronpa by PALM, and without the presence of gp41 (Fig. S5I). Our imaging confirmed the staining of pTCRζ in clusters of TCRζ-Dronpa at the nanoscale, beyond their colocalization in clusters that was evident by diffraction-limited microscopy (Fig. 6A). See SI for further details on PALM, dSTORM and FRET imaging.

### Data availability

Data supporting the findings of this study are available from the corresponding author upon reasonable request.

## Electronic supplementary material

Supplementary Information

Movie M1

Movie M2
