# Supplementary material for: Gp41 dynamically interacts with the TCR in the immune synapse and promotes early T cell activation
Source: Sci Rep. 2018 Jun 27;8:9747. doi: 10.1038/s41598-018-28114-5 (PMC6021400; doi:10.1038/s41598-018-28114-5)

**Supplemental Information**

# Gp41 dynamically interacts with the TCR in the immune synapse and promotes early T cell activation

Oren Yakovian[1], Roland Schwarzer[2], Julia Sajman[1], Yair Neve-Oz[1], Yair Razvag[1], Andreas Herrmann[2], Eilon Sherman[1*]

*[1]Racah Institute of Physics, The Hebrew University, Jerusalem, Israel, 91904 [2]Department of Biology, Molecular Biophysics, Humboldt Universität zu Berlin, 10115 Berlin, Germany.*

** Corresponding author*

Correspondence to: Eilon Sherman (sherman@phys.huji.ac.il)

## Materials and Methods

**Cloning.** Proteins tagged with the photoactivatable fluorescent proteins (PAFPs) Dronpa (MBL International Corporation), PAmCherry and PAGFP were generated in EGFP-N1 or EGFP-C1 vectors (Clontech). PAFPs genes were cut from a vector through digestion with restriction enzymes (typically AgeI and NotI, XbaI or BsrGI). PAFP genes then served to replace existing fluorescent proteins (FP) in previously used constructs [1,2] using similar digestion reactions and ligation of the PAFP insert (using Thermo Scientific Ligase T4). Validation of cloning was done by DNA sequencing of the inserts. Specifically, newly generated constructs for this study included: gp41(JRFL)ΔED*-PAmCherry, gp41(JRFL)ΔED*-PAGFP, gp41(JRFL)ΔED*-mCRAC-Dronpa, gp41(JRFL)ΔED*-mCRAC-PAmCherry, gp41(HXB2)ΔED*-PAmCherry, gp41(HXB2)-PAmCherry, gp41(HXB2)-mTMD-PAmCherry, and GPI-PAmCherry. The previously developed construct TCRζ-Dronpa was already available for this work from previous studies [1].

**Jurkat T cell cultures and transfections**

E6.1 Jurkat T cells [3] were grown in Complete medium: PRMI (Roswell Park Memorial Institute medium), 10% Fetal Bovine Serum, 1% Pen/Strep. The cells were grown at 37 °C in a humidified $CO_2$ incubator. A stable cell line expressing TCRζ-Dronpa was maintained with Geneticin at 1.5mg/ml (G418, Invitrogen) and was available for this study from previous published studies [4,5]. E6.1 cells or the TCRζ stable cells were transfected by a Neon electroporation system (Invitrogen) for the transient transfection of fluorescent proteins. For transfection, desired pulse conditions were set on the device based on our cell type and an optimization of the parameters. The chosen transfection parameters for this study were: Voltage – 1325 V, Pulse width – 15 msec and the number of pulses was 3. After transfection, cells were seeded into prepared 96-well plates with 0.1 ml of culture medium without antibiotics. After 24-48 hours of incubation at 37 °C cells were ready for imaging. Transiently transfected cells were maintained in transfection medium, monitored for positive expression of PAmCherry, Dronpa or PAGFP chimeras and imaged within 48-72 hours from transfection.

**Sample preparation.** The preparation of coverslips for imaging spread cells followed a previously described technique [6]. Briefly, for PALM imaging, we used #1.5 glass 4 well-chambers (LabTek or Ibidi). The chambers were washed with 1M HCL in 70% EtOH, ~0.5 ml per chamber at room temperature for 10 min. The liquid was then aspirated and chambers were dried at 37 $^{0}$C for 1 hour. These glass chambers were then coated with 0.01% poly-L-lysine (Sigma, P4707) diluted in water at room temperature for 15 min. Liquid was aspirated and chambers were dried at 37 $^{0}$C for 2 hours. In the next step, glass chambers were coated with 0.4 ml 10μg/ml mouse αhuman CD3 (Biotest, 16-0038-85) or mouse αhuman CD45 (Bactlab Diagnostics, PMG555480) or mouse αhuman CD11a/LFA (BD Pharmingen, 555378) antibody diluted in phosphate-buffered saline (PBS). Finally, chambers were washed with PBS 3 times. Antibody-coated chambers were stored at 4 $^{0}$C with 0.5 ml PBS in a humidity chamber. Before imaging, cells were resuspended in imaging buffer (RPMI without phenol red, 10% FBS, 25mM Hepes) at a concentration of $1x10^{6}$/150 μl and 100,000-500,000 cells were dropped onto glass chambers for PALM imaging. Cells were incubated at 37 $^{0}$C for a desired spreading time (typically 3-5 min) and fixed with 4% Paraformaldehyde (PFA) for 30 min at 37 $^{0}$C. As a last step of preparation for imaging fixed cells, the glass chambers were washed with PBS 3 times. For live cell imaging, we kept the cells in imaging buffer in an incubator (37 $^{0}$C), and dropped them into wells containing imaging buffer as they were mounted on the microscope.

**Single molecule localization microscopy.** Two-colour photoactivated localization microscopy (PALM) imaging was conducted similarly to the imaging previously described [1], using a total internal reflection (TIRF) Nikon microscope. In this system, excitation of the photoactivatable fluorescent proteins (PAFPs) was achieved with a 488 nm laser line for PAGFP and Dronpa, and a 561 nm laser line for PAmCherry. Photoactivation of the PAFPs was performed with a 405 nm laser line. Emission was detected by an Ixon$^{+}$ Ultra EMCCD

(Andor) mounted on the microscope. PALM images were analysed using an algorithm to identify peaks in individual frames and group them into functions that reflect the positions of single molecules [7]. Sample sizes were chosen to account for cell to cell variability, within experimental constraints.

For direct Stochastic Optical Reconstruction Microscopy (dSTORM) combined with PALM, or for diffraction limited microscopy, cells stably expressing TCRζ-Dronpa were transfected with a gp41(JRFL)ΔED*-PamCherry plasmid. The cells were dropped and fixed on αCD3ε antibody coated coverslip, as described above. For permeabilization, we added 0.4 ml 0.1% Triton X-100 in PBS per well and incubated for 3-4 minutes. The cells were blocked by 2% normal goat serum in PFN (PBS + 10% serum + 0.02% sodium azide) for 30 minutes. For 0.5 million cells, we added 0.5 μg rabbit αhuman phsophoTCRζ (pY83 of TCRζ; Thermo Scientific, 700177) as a primary antibody diluted in 2% normal goat serum in PFN, incubated for 60 minutes at RT and washed 3 times with PFN. Alexa647 was added as a secondary antibody (αrabbit) diluted (1/3000) in 2% normal goat serum in PFN, incubated for 45 minutes at RT and washed 3 times with PFN.

Förster Resonance Energy Transfer (FRET) imaging was conducted using TIRF Nikon microscope. E6.1 cells were resuspended in (0.5 million cells in 100 μl) a FACS buffer (10%FBS, 0.02% sodium azide in PBS) and incubated on ice for 10 minutes. The primary antibody, 0.5 μg mouse αhuman CD3ε-Alexa647 (R&D Systems, FAB100R) as acceptor and 0.5 μg rabbit αhuman αGFP-Alexa555 (Invitrogen, A31851) as donor, were added and incubated for 30 minutes on ice. The cells were washed 3 times with PBS and resuspended in 0.5 ml imaging buffer. These cells were dropped and fixed on αCD45 antibody coated coverslip, as described above. For negative control, αGFP-Alexa555 was replaced by αCD45 and αmouse-Alexa555 and the cells were dropped on αCD11a/LFA-coated coverslip. Note that the staining of CD45 here prevented the use of αCD45 antibody coated coverslip for this sample. A positive control was performed using an αCD3ε-Alexa647 primary antibody and a secondary antibody carrying αmouse Alexa555 that targeted the αCD3ε-Alexa647 primary antibody.

**Flow cytometry.** For flow cytometry Jurkat E6.1 cells were transfected with a gp41 (JRFL)ΔED*-YFP or gp41(HXB2)-YFP plasmid. After 24 hours, 0.5 μg/ml mouse αhuman CD3 was added to stimulate for 3 hours. The cells were resuspended in (0.5 million cells in 100 μl) a FACS buffer and incubated on ice for 10 minutes. The primary antibody, 0.5 μg mouse IgG2a αhuman CD69 (abcam, MM0688-14F49) was added and incubated for 30 minutes on ice. The cells were washed 2 times with FACS buffer and PBS. Goat anti-mouse IgG2a Alexa647 (Thermo Scientific, A21241) was added as a secondary antibody diluted (1/3000) in FACS buffer, incubated for 30 minutes at RT and washed 2 times with FACS buffer and PBS. For negative controls for the flow cytometry data (Fig. 7A,B), we employed mock transfections without any vector or with an empty vector, which yielded similar results.

Cell death analyses were performed using APC-Annexin V (640919, BioLegend) for apoptosis detection and PI (P4864, Sigma) for necrosis detection prior FACS analyses. Staining was done according to manufacturer protocol.

## Analyses

**PALM rendering** - movies generated by PALM imaging were analysed by the ThunderSTORM software [8] for the identification of individual peaks in the movie frames. Next, peaks were grouped and assigned to individual molecules for rendering of the PALM images. Peak grouping used a distance threshold and a temporal gap to account for possible molecular blinking [9]. A range of temporal gaps were considered for each fluorophore separately in order to minimize possible over-counting of molecules. Individual molecules are presented in PALM and dSTORM images with intensities that correspond to the probability density values of their fitted Gaussian with respect to the maximal probability density values detected in the field.

**Live cell movie.** Two-colour PALM imaging of a live Jurkat E6.1 cell expressing TCRζ-Dronpa and gp41(JRFL)ΔED*-PAmCherry on an αCD3-coated coverslips. Each of the images was collected from 50 frames at a frame rate of 13.1 fps, yielding an effective frame time of 3.8s. The PALM movie is shown in Movie M1. A filtered movie (Movie M2) was generated by filtration of the TCR (green) channel using a Kalman filter (ImageJ), with variance estimate of acquisition noise of 0.05 and bias of the prediction of 0.8.

**Second order statistics and pair correlation function**

PALM and dSTORM imaging result in a point pattern that marks the centre locations of single molecules. To describe the density of the point pattern as output microscopy imaging, and explain the mechanism responsible for the measured organization of the molecules, first order statistics is not sufficient, and we turned to second-order statistics, as explained below. Additionally, if the pattern refers to two or more different populations, bivariate second order statistics is necessary to explore the correlation behaviour of the populations. The second order characteristics depend only on the distance r between points, but not on the direction or the location of points for a homogeneous and isotropic point pattern. Pair correlation function (PCF, denoted here also as *g(r)*) describes and quantifies in a point pattern how density varies as a function of distance from a reference particle. Usually the PCF is normalized by the density of the sample. Univariate PCF can be used to explore a point pattern of a single species. Bivariate PCF as defined below (Eqs. 2,4) quantifies for two species, the density of pattern 2 at distance r from an arbitrary point of pattern 1.

An appropriate geometry is therefore to adopt circular shapes such as the circles of Ripley’s K-function or the rings of Wiegand-Moloney's O-ring statistic as a basis for the spatial statistics [10]. Using rings instead of circles has the advantage that one can isolate specific distance classes, whereas the cumulative K-function confounds effects at larger distances with effects at shorter distances. In this work, we used O-ring statistic because it can detect aggregation or dispersion at a given distance r whereas K-function can detect aggregation or dispersion up to a given distance r. For two point patterns that represent two different populations, the bivariate *K*-function $K_{12}(r)$ is defined as [10]:

(1) $\boldsymbol{k_{12}(r) = \lambda_2^{-1}}$ [the number of points of pattern 2 in a distance≤ from an arbitrary point of pattern 1]

$\boldsymbol{r}$ where $\boldsymbol{\lambda_2}$ is the mean areal density of points of pattern 2 [10].

The bivariate PCF $g_{12}(r)$ is defined in a similar fashion to Ripley's K-function apart from replacing the circles of radius r with rings of radius $r$:

(2) $$\boldsymbol{g_{12}(r) = \lambda_2^{-1} \begin{bmatrix} \textbf{the number of points of pattern 2 at distance } r \\ \textbf{from an arbitrary point of pattern 1} \end{bmatrix}}$$

And Wiegand-Moloney's $O_{12}(r)$ is defined by [10]:

(3) $$\boldsymbol{O_{12}(r) = \lambda_2\, g_{12}(r)}$$

In this study we used the bivariate functions in order to indicate attraction between the two patterns up to distance $r$. For a single population pattern, the univariate $g(r) \equiv g_{11}(r)$ is used in analogy to the $g_{12}(r)$ function, by replacing points of pattern 2 by points of pattern 1. Univariate analysis indicates the conjugation to be together and self-clustering of a single population pattern [10].

In this step we calculated the PCFs and compared them to different models using Monte-Carlo simulations. For that, we used a custom code in Matlab. Two useful models for the evaluation of the organization and extent of interactions between species are described below.

Following a similar notation to Wiegand [10], a bivariate PCF can be calculated for a pixelated image using the following definitions:

(4) $$g_{12}(r) = \frac{A}{n_2} \frac{\frac{1}{n_1}\sum_{k=1}^{n_1} Pnts\left[S_2, R_{1,k}^{w}(r)\right]}{\frac{1}{n_1}\sum_{k=1}^{n_1} Area\left[R_{1,k}^{w}(r)\right]}$$

where, $R_{1,k}^{w}(r)$ is the ring with radius $r$ and width $w$ centred on the $k$'th point of type $2$ (here points of type $2$ are simply type $i$ molecules, or $S_2$, as defined above). $n_i$ is the total number

of points of type $i$ in the study region of area $A$. The operator *Pnts* $[S_j,X]$ counts the points of type $j$, namely $S_j$, in region $X$. The operator *Area* counts the number of cells in the region $X$.

### Complete Spatial Randomness (CSR)

The simplest way to quantify the deviation of a point pattern from randomly distribution is comparison to a Complete Spatial Randomness (CSR) distribution. CSR is a Poisson and homogeneous process. In other words, CSR assumes no interaction between the points of the pattern and has a constant density as the first order statistics over the study region. The difference between this model and the point pattern of the sample can be observed by comparison of their PCFs [10]. By default, CSR of two different species would result in flat PCFs with a value of 1 that indicate no interaction (i.e. no spatial correlation) between the species. Note that there many alternative models (with orthogonal processes of the different species) could give rise to no interaction.

### The Random Labelling model

In order to investigate whether or not two species in a joint point pattern are significantly interacting, we used the random labelling (RL) model. In this model, points of pattern 1 ($n_1$) and points of pattern 2 ($n_2$) distribute randomly on $n_1$+ $n_2$ fixed locations. Multiple Monte-Carlo simulations replicate nineteen times the point patterns while randomly re-labelling the points (with the number of points from each species). The bivariate PCF of the original point patter $g_{12}$(r) is then compare to the bivariate PCFs of the simulations. We used the lowest and highest $g_{12}$(r) of the different simulations as a 95% confidence interval for the acceptance or rejection of the model as a null hypothesis. Agreement of the data with the RL model indicates homogeneous mixing, and hence strong interaction (in a statistical sense) of the two species under study. Prior knowledge on the physical binding of the two species (e.g. from biochemical assays) can then help to interpret the studied interactions as physical binding events of the species.

### Comparing bivariate PCFs of multiple cells (or realizations)

The RL model is individual to each cell (or point pattern realization), and thus there is no straight forward way to compare bivariate PCFs (BPCFs) from multiple cells. In this study, we propose and apply two complementary ways to compare and average bivariate PCFs computed for multiple cells: (i) the extent of mixing (EOM), and (ii) the standardized bivariate PCF (SBPCF).

(i) **The extent of mixing (EOM)**

In previous studies, we have introduced a measure we termed the 'Extent of Mixing' [1,11]. For convenience, we briefly introduce this measure in Fig. S2A and below. This measure is first computed individually for each cell as follows:

(5) $$EOM^{i}(r) = \frac{g_{12}^{i}(r) - 1}{g_{12}^{RL,i}(r) - 1}$$

where $g_{12}^{i}(r)$ is the BPCF for cell *i*, and $g_{12}^{RL,i}(r)$ is the average of the nineteen simulated BPCFs due to the RL model for cell *i*. Meaning, we take the height of the BPCF above the baseline of the model of no-interaction (at a specific length scale) and divide it by the height of the average BPCF due to the RL model. Next, the EOM can be readily averaged for multiple (N) cells, following Eq. 6:

(6) $$EOM(r) = \frac{1}{N}\sum_{i=1}^{N} EOM^{i}(r)$$

The errors are computed as:

(7) $$SEM(r) = \sqrt{\frac{1}{N^{2}}\sum_{i=1}^{N} EOM(r)^{2}}$$

**(ii) Standardized bivariate PCF (SBPCF)**

To further compare molecular interaction within multiple cells, we took a second approach, by defining a standardized version of the BPCFs (SBPCF), $\hat{g}_{12}{}^{i}(r)$, independently for each cell $i$ following Eq.8.

(8) $$\hat{g}_{12}{}^{i}(r) = \frac{g_{12}^{i}(r) - \left\langle \tilde{g}_{12}^{i}(r) \right\rangle}{\tilde{\sigma}^{i}(r)}$$

where, $\tilde{g}_{12}{}^{i}(r)$ is the set of all simulated $g_{12}{}^{i}(r)$ for cell $i$ (i.e. the previous notation $g_{12}^{RL,i}(r)$ in Eq. 5 is now replaced with the more elaborate term $\left\langle \tilde{g}_{12}^{i}(r) \right\rangle$). We further define:

(9) $$\tilde{\sigma}^{i}(r) = \max\left(\left|\left(\tilde{g}_{12}^{i}(r) - \left\langle \tilde{g}_{12}^{i}(r) \right\rangle\right)\right|\right)$$

Here, $2\tilde{\sigma}^{i}(r)$ denotes the 95% confidence interval of the BPCF of cell $i$ due to the nineteen simulated random sets of the null hypothesis. We then took the average of the standardized conditional bivariate PCFs and the standard error of the mean (SEM) over multiple $N$ cells using the following equations (Eqs. 10 and 11):

(10) $$\left\langle \hat{g}_{12}(r) \right\rangle = \frac{1}{N} \sum_{i=1}^{N} \hat{g}_{12}^{i}(r)$$

(11) $$SEM(r) = \sqrt{\frac{1}{N^{2}} \sum_{i=1}^{N} \hat{g}_{12}^{i}(r)^{2}}$$

**Conditional bivariate PCF –** Recently, we have shown how 3 colour SMLM imaging could help to quantify synergy in the interactions of 3 interacting species [5]. We briefly describe this analysis below, while a comprehensive discussion and robustness analyses of this statistics can be found in this previous publication [5]. The conditional bivariate statistics and synergy analyses rely on a first step of choosing a subpopulation of one species, based on its proximity to (or interaction with) a second species. Then, the interaction of this subpopulation with a third species can be compared to a similar interaction, yet for random

sets of the first species. This analysis is applied to study regions that cover most of the apparent footprint of the cells. As a next step, the standardization of these conditional bivariate PCF curves allows for their comparison and for their averaging over multiple cells. After this step, significant synergy in molecular interactions can be detected.

We briefly describe below the algorithm by referring to an example where we study the interactions between type 2 and type 3 molecules, upon their binding to type 3 molecules. The set of x,y coordinates of each molecular species is denoted here by $S_i$, where $i$ = 1,2,3. First, we identified the proximity of molecules from two species of interest (e.g., Type 2 and Type 3) using the Boolean function Pr (Eq. 12). Pr is calculated for each pair of molecules ($s_2$, $s_3$) from $S_2$ and $S_3$, as follows:

(12) $$\Pr(s_2, s_3) = \begin{cases} 1 & Dist(s_2, s_3) \le d_{th} \\ 0 & Dist(s_2, s_3) > d_{th} \end{cases}$$

where the operator $Dist(s_i,s_j)$ is the Euclidean distance between the points $s_i$ and $s_j$ and where $d_{th}$ is the threshold for defining proximity. The value of this threshold, $d_{th}$, was varied between 20 nm and 60 nm to check the robustness of our analyses approach (as in [5]). A threshold of 36 nm was then chosen to select the interacting Type 2 molecules, using the function Pr to obtain a subset $S_2'$ of the points of type 2 (namely $S_2$), following the set-builder notation of Eq. 13. Notably, in our SMLM measurements the localization uncertainty of individual molecules peaked at ~25nm for all colours. Thus, the 36 nm threshold was about the rms size of the uncertainty of two colocalized molecules in our study. Another consideration for setting the proximity threshold involves the molecular density in the data (as detailed in [5]).

(13) $$S_2' = \{ s_2 \mid \exists s_3 \in S_3 \quad such \quad that \quad \Pr(s_2, s_3) = 1 \}$$

Together, Eqs. 3 and 4 state that $s_2$ is included in $S'_2$ if there exists at least one proximal molecule $s_3$ from $S_3$ that lies below the threshold distance $d_{th}$ from $s_2$.

Next, we calculated the *conditional* bi-variate pair-correlation function (PCF; $g_{12|\Pr(2,3)}(r)$) of the selected subset of Type 2 molecules, $S_2'$, with a third molecular species of Type 1.

Similar to the BPCF (Eq. 4), the *conditional* bi-variate pair-correlation function (PCF; $g_{12|\Pr(2,3)}(r)$) is defined in Eq. 14. This equation now refers to $S_2'$, the proximity-selected sub-population of $S_2$, with a total number of molecules of $n_{2'}$.

$$(14) \qquad g_{12|\Pr(2,3)}(r) = \frac{A}{n_{2'}} \frac{\frac{1}{n_1}\sum_{k=1}^{n_1} Pnts\left[S_2', R_{1,k}^{w}(r)\right]}{\frac{1}{n_1}\sum_{k=1}^{n_1} Area\left[R_{1,k}^{w}(r)\right]}$$

To check the significance of the interaction synergy, the conditional bivariate PCF was compared to the bivariate PCF of Type 1 and the same number of chosen Type 2 molecules that were randomly spread across the positions of all of the identified type 2 molecules, regardless of their proximity to Type 1 molecules (yielding $\tilde{g}_{12}(r)$ in Eqs. 15 and 16 below). A Monte-Carlo simulation was used in this later stage to generate nineteen control sets through the described random placement of molecules and to mark a range of 95% confidence interval, within which the interaction is not significantly synergic.

As mentioned earlier, to further compare the synergy of the molecular interaction within multiple cells, we first standardized the conditional bi-variate PCFs independently for each cell *i* following Eq.15.

$$(15) \qquad \hat{g}_{12|\Pr(2,3)}{}^{i}(r) = \frac{g_{12|\Pr(2,3)}^{i}(r) - \left\langle \tilde{g}_{12}^{i}(r) \right\rangle}{\tilde{\sigma}^{i}(r)}$$

where,

(16) $$\tilde{\sigma}^i(r) = \max\left(\left|\left(\tilde{g}^i_{12|\Pr(2,3)}(r) - \left\langle \tilde{g}^i_{12}(r) \right\rangle\right)\right|\right)$$

As before (Eq. 9), $2\tilde{\sigma}^i(r)$ denotes the 95% confidence interval of the bivariate PCF of cell *i* due to the nineteen simulated random sets of the null hypothesis. In analogy to Eqs. 10,11, we then took the average of the standardized conditional bivariate PCFs and the standard error of the mean (SEM) over multiple *N* cells using the following equations (Eqs.17 and 18):

(17) $$\left\langle \hat{g}_{12|\Pr(2,3)}(r) \right\rangle = \frac{1}{N}\sum_{i=1}^{N} \hat{g}^i_{12|\Pr(2,3)}(r)$$

(18) $$SEM(r) = \sqrt{\frac{1}{N^2}\sum_{i=1}^{N} \hat{g}^i_{12|\Pr(2,3)}(r)^2}$$

Finally, to report on the synergy of interactions due to multiple cells, the resultant curves of $\left\langle \hat{g}_{12|\Pr(2,3)}(r) \right\rangle$ and SEMs were plotted (red line and error bars in Fig. 6F) along with the normalized 95% confidence interval (dotted gray lines in Fig. 6F). The departure of the averaged PCF from the confidence intervals serves to reject the null hypothesis, indicating that the binding of Type 1 and Type 2 is promoted by the interaction of Type 2 molecules with molecules of Type 3.

All calculations and graphical rendering of PCFs were conducted using a published software [10] or custom codes written in Matlab (MathWorks).

**FRET imaging and analyses –** Here, we employed the donor-sensitized acceptor fluorescence for FRET imaging of the interaction between the TCR and gp41(JRFL)ΔED*-PAGFP. For such imaging, three imaging channels can be defined: (i) a donor channel, optimized for the donor fluorescence of Alexa555, using 561nm excitation and detecting its

emission using ET 600/50 M emission filter; (ii) an acceptor channel, optimized for the acceptor emission of Alexa647, using 647nm excitation and ET 700/75 M emission filter; (iii) a FRET channel, using 561nm excitation (as for the donor) and emission detection between ET 700/75 M (as for the acceptor). Sensitized FRET involves crosstalk between the imaged channels due to direct excitation of the acceptor at the donor excitation and bleed-through of the donor emission to the acceptor and FRET channels. To correct for these cross-talks, one first subtracts the background in all channels, yielding fluorescence signal for channels of the donor ($F_D$), acceptor ($F_A$) and FRET ($F_F$). Next, we defined the following cross-talk factors [12]:

$$(19) \qquad S_1 = \frac{F_F}{F_D}, \quad S_2 = \frac{F_F}{F_A}, \quad S_3 = \frac{F_A}{F_D}, \quad S_4 = \frac{F_D}{F_A}$$

Averaging over multiple donor-only samples yielded $S_1$=0.110 and $S_3$=0.003. Similarly, acceptor-only samples yielded $S_2$=0.082 and $S_4$=0.003. (Note that, as expected, $S_3$ and $S_4$ are essentially negligible).

Next, the rate of relative detection sensitivity of the excited acceptor compared to the excited donor is described by another factor, α, as follows:

$$(19) \qquad \alpha = \frac{I_{FinA}}{I_{DinD}} \cdot \frac{L_D}{L_A} \cdot \frac{B_D}{B_A} \cdot \frac{\varepsilon_D}{\varepsilon_A}$$

Where $I_x$ is the fluorescence intensity, $L_x$ is the labelling ratio, $B_x$ is the antigen ratio, and $\varepsilon_x$ is the extinction coefficient at maximal donor excitation, and the subscript $_x$ denoted either the donor (D) or the Acceptor (A). The manufacturer specifies $L_{D,A}$ = 3 for both the donor and the acceptor, which we further confirmed by spectral absorption measurements. We also consider $B_D$=$B_A$, while ε values for the donor and acceptor were taken from manufacturer specifications. These parameters yielded $\alpha$=0.68.

The energy transfer efficiently per donor excitation can now be determined as [12]:

(19) $$E = \frac{A}{1+A}$$

Where

(19) $$A = \frac{F_F - F_D \cdot S_1 - F_A \cdot S_2}{\alpha \cdot F_D}$$

To increase the reliability of the calculations and to prevent low-level noise from distorting the calculated FRET efficiency, we excluded pixels below half of the intensity of the average background across the imaging field or 0.025 of the maximal intensity throughout the imaging field. We also avoided saturation throughout the imaging, and set the value of saturated pixels to 0. FRET images were rendered in pseudo-colour with a Fire colour map. All calculations were done using a custom Matlab code.

To estimate the significance of our results, we report FRET for 14 cells from 2 replicate experiments. As described above, we also generated a negative control sample by staining with the donor and the acceptor labelled antibodies two proteins that are not interacting (CD3ε and CD45). A positive control sample was generated by targeting the donor labelled antibody as a secondary to an acceptor labelled αCD3ε antibody.

## References

1 Sherman, E., Barr, V., Manley, S., Patterson, G., Balagopalan, L., Akpan, I., Regan, C. K., Merrill, R. K., Sommers, C. L., Lippincott-Schwartz, J. & Samelson, L. E. Functional nanoscale organization of signaling molecules downstream of the T cell antigen receptor. *Immunity* **35**, 705-720, (2011).

2 Schwarzer, R., Levental, I., Gramatica, A., Scolari, S., Buschmann, V., Veit, M. & Herrmann, A. The cholesterol-binding motif of the HIV-1 glycoprotein gp41

regulates lateral sorting and oligomerization. *Cellular microbiology* **16**, 1565-1581, (2014).

3 Schneider, U., Schwenk, H. U. & Bornkamm, G. Characterization of EBV-genome negative "null" and "T" cell lines derived from children with acute lymphoblastic leukemia and leukemic transformed non-Hodgkin lymphoma. *International journal of cancer. Journal international du cancer* **19**, 621-626, (1977).

4 Neve-Oz, Y., Razvag, Y., Sajman, J. & Sherman, E. Mechanisms of localized activation of the T cell antigen receptor inside clusters. *Biochim Biophys Acta* **1853**, 810-821, (2015).

5 Sherman, E., Barr, V. A., Merrill, R. K., Regan, C. K., Sommers, C. L. & Samelson, L. E. Hierarchical nanostructure and synergy of multimolecular signalling complexes. *Nature communications* **7**, 12161, (2016).

6 Bunnell, S. C., Barr, V. A., Fuller, C. L. & Samelson, L. E. High-Resolution Multicolor Imaging of Dynamic Signaling Complexes in T Cells Stimulated by Planar Substrates. *Sci. STKE* **2003**, pl8, (2003).

7 Rust, M. J., Bates, M. & Zhuang, X. Sub-diffraction-limit imaging by stochastic optical reconstruction microscopy (STORM). *Nat Methods* **3**, 793-795, (2006).

8 Ovesny, M., Krizek, P., Borkovec, J., Svindrych, Z. K. & Hagen, G. M. ThunderSTORM: a comprehensive ImageJ plug-in for PALM and STORM data analysis and super-resolution imaging. *Bioinformatics* **30**, 2389-2390, (2014).

9 Betzig, E., Patterson, G. H., Sougrat, R., Lindwasser, O. W., Olenych, S., Bonifacino, J. S., Davidson, M. W., Lippincott-Schwartz, J. & Hess, H. F. Imaging intracellular fluorescent proteins at nanometer resolution. *Science* **313**, 1642-1645, (2006).

10 Wiegand, T. & Moloney, K. A. Rings, circles, and null-models for point pattern analysis in ecology. *Oikos* **104**, 209-229, (2004).

11 Sherman, E., Barr, V. A. & Samelson, L. E. Resolving multi-molecular protein interactions by photoactivated localization microscopy. *Methods* **59**, 261-269, (2013).

12 Nagy, P., Vamosi, G., Bodnar, A., Lockett, S. J. & Szollosi, J. Intensity-based energy transfer measurements in digital imaging microscopy. *European biophysics journal : EBJ* **27**, 377-389, (1998).

## Supplemental Figure Legends

**Table S1. Centre vs. periphery recruitment and clustering of gp41 at the IS**

**Table S2. The effect of gp41 expression on cell killing**

**Fig. S1. Gp41 constructs used in the study for single molecule localization microscopy**

(A) Localization uncertainty of the fluorophores used in this study for single molecule localization microscopy by either PALM or dSTORM. (B) The gp41 constructs used in this study are presented according to their domains. Red highlights mark a mutation or replacement. Black text marks constructs originated from the JRFL isolate, while red text marks constructs originated from the HXB2 isolate. (C) Sequence alignment of the constructs from the JRFL (Uniprotentry: Q75760) and HXB2 (P04578) HIV-1 virus isolates.

**Fig. S2. Bivariate second order statistics and SBPCF curves of the molecular interactions under study**

(A)(left) A representative bivariate pair correlation function (PCF) is shown in blue. This curve is typically compared to two models – a model of no interaction (black line, with a value of 1) and a model of random labelling (RL). The RL model is simulated by generating 19 random sets where the label of the species is randomly assigned without changing their detected positions. The maximum and minimum of these simulations at each length-scale yield a 95% confidence interval of this model (black dotted lines). Importantly, this curve is shown for a single cell and cannot be averaged over multiple cells since the RL simulations are individual to the presented cell. (middle) The standardized bivariate PCF (SBPCF) is introduced by Eqs. 8 and 9 in the SI. It is the difference between the heights of the measured bivariate PCF and the average of the simulations of the RL model, normalized by the width of the 95% confidence interval of the RL model. Thus, the SBPCF curve (blue line) in the right graph corresponds to the PCF curve (blue line) in the left graph. The RL upper and lower boundaries of the RL confidence interval take values of +1 and -1, respectively. The no-interaction model becomes the lowest negative curve (black line). Relations between the corresponding curves of the two graphs are highlighted by blue and black arrows.

Importantly, the SBPCF curves can now be averaged over multiple cells, yielding the errors (SEM) at each length-scale. (right) The extent of mixing (EOM) is defined as the ratio of the height of the PCF curve in comparison to the average PCF height of the simulated RL model (see definitions by Eqs. 5 and 6).

(B-L) SPCF curves are shown for the specified constructs (blue), compared to the 95% confidence interval of a Random labelling model (black dotted lines at 1 and -1) and a model of no interaction (NI; black solid line) for: (B) TCRζ-Dronpa and gp41(JRFL)ΔED*-PAmCherry on αCD45 (for the data in Fig. 1A-C). (C) TCRζ-Dronpa and gp41(JRFL)ΔED*-PAmCherry on αCD3 (for the data in Fig. 1D-F). (D) gp41(JRFL)ΔED*-PAGFP and GPI-PAmCherry on αCD3+CD45 (for the data in Fig. 3A-C). (E) Gp41(JRFL)-mCRAC-Dronpa and GPI-PAmCherry on αCD3+CD45 (for the data in Fig. 3G-F). (F) Gp41(JRFL)ΔED*RFL)-PAGFP and gp41(JRFL)ΔED*-mCRAC-PAmCherry on αCD3 (for the data in Fig. 3G-I). (G) TCRζ-Dronpa and gp41(JRFL)ΔED*-mCRAC-PAmCherry on αCD3 (for the data in Fig. 3J-L). (H) TCRζ-Dronpa and gp41(HXB2)-PAmCherry on αCD3 (for the data in Fig. 4A-C). (I) TCRζ-Dronpa and gp41(HXB2)-PAmCherry on αCD45 (for the data in Fig. 4D-F). (J) TCRζ-Dronpa and gp41(HXB2)-PAmCherry on αCD3 (for the data in Fig. 5A-C). (K) TCRζ-Dronpa and gp41(HXB2)-PAmCherry on αCD45 (for the data in Fig. 5D-F). (L) TCRζ-Dronpa and gp41(HXB2)-mTMD-PAmCherry on αCD3 (for the data in Fig. 5G-I). Error bars are SEM.

**Fig. S3. Negative controls for gp41-TCR interaction**

(A) Two-colour PALM imaging of fixed E6.1 Jurkat cells expressing TCRζ-Dronpa and gp41(JRFL)ΔED*-PAmCherry on coverslips coated with αCD11. Cells were dropped and let spread on the coverslip for 3 min before fixation. Bars – 2 μm (left) and 200 nm (right).

Shown is a representative cell (N=6). (B) PCF of TCRζ-Dronpa (green) and gp41(JRFL)ΔED*-PAmCherry (red). (C) EOM of TCRζ-Dronpa and gp41(JRFL)ΔED*-PAmCherry.

(D) Two-colour PALM imaging of fixed E6.1 Jurkat cells expressing Syntaxin1A-Dronpa and gp41(JRFL)ΔED*-PAmCherry on coverslips coated with αCD45. Cells were dropped and let spread on the coverslip for 3 min before fixation. (E) PCF of Syntaxin1A-Dronpa and gp41(JRFL)ΔED*-PAmCherry (red). (N=7). (F) EOM of Syntaxin1A-Dronpa and gp41(JRFL)ΔED*-PAmCherry.

(G) Two-colour PALM imaging of fixed E6.1 Jurkat cells expressing Syntaxin1A-Dronpa and gp41(JRFL)ΔED*-PAmCherry on coverslips coated with αCD3. Cells were dropped and let spread on the coverslip for 3 min before fixation. (H) PCF of Syntaxin1A-Dronpa and gp41(JRFL)ΔED*-PAmCherry (red). (N=15). (I) EOM of Syntaxin1A-Dronpa and gp41(JRFL)ΔED*-PAmCherry.

(K) Single-colour PALM imaging of fixed Jurkat E6.1 cells expressing TCRζ-Dronpa on coverslips coated with either αCD3 (left) or αCD45 (middle). Cells were dropped and let spread on the coverslip for 3 min before fixation. PCF of TCRζ-Dronpa (N=30 for each condition).

**Fig. S4. Imaging the TCR and gp41 in cell cross-sections**

(A) Confocal imaging of fixed E6.1 Jurkat cells expressing TCRζ-Dronpa and gp41(HXB2)-PAmCherry on an αCD3-coated coverslips. Cells were dropped and let spread on the coverslip for 3 min before fixation. Bar – 2 μm (left). Shown is a representative cell (N=3). (B) Two-colour PALM imaging of fixed E6.1 Jurkat cells expressing TCRζ-Dronpa and gp41(HXB2)-PAmCherry on an αCD3-coated coverslips, prepared for imaging as in A. The

cells were imaged using TIRF or epi-illumination while focusing at different heights from the coverslip (0 μm) to ~2μm and ~4μm above the coverslip (upper, middle and lower rows, respectively). Bar – 2 μm (left). Shown is a representative cell (N=6).

**Fig. S5. Controls of molecular labelling for PALM and dSTORM**

(A) Two-colour PALM imaging of fixed E6.1 Jurkat cells expressing TCRζ-PAmCherry and gp41(JRFL)ΔED*-PAGFP on an αCD3-coated coverslips. Cells were dropped and let spread on the coverslip for 3 min before fixation. Bars – 2 μm (left) and 200 nm (right). Shown is a representative cell (N=3). (B) PCF of gp41(JRFL)ΔED*-PAGFP (green) and TCRζ-PAmCherry (red). (C) Standardized bivariate PCF of gp41(JRFL)ΔED*-PAGFP and TCRζ-PAmCherry (blue), compared to the 95% confidence interval of a Random labelling model (black dotted lines at 1 and -1) and a model of no interaction (NI; black solid line). (D) The extent of mixing of gp41(JRFL)ΔED*-PAGFP and TCRζ-PAmCherry, calculated for each wide area image in A.

(E) Two-colour PALM imaging of fixed E6.1 Jurkat cells expressing gp41(JRFL)ΔED*-PAmCherry and gp41(JRFL)ΔED*-PAGFP on an αCD3-coated coverslips. Cells were dropped and let spread on the coverslip for 3 min before fixation. Middle image shows a zoomed area with rendering of detected molecules using overlapping Gaussians, while the right image shows the same zoomed area using scattered dots. Bars – 2 μm (left) and 200 nm (middle and right). Shown is a representative cell (N=3). (F) PCF of gp41(JRFL)ΔED*-PAGFP (green) and gp41(JRFL)ΔED*-PAmCherry (red). (G) Standardized bivariate PCF of gp41(JRFL)ΔED*-PAGFP and gp41(JRFL)ΔED*-PAmCherry (blue), compared to the 95% confidence interval of a Random labelling model (black dotted lines at 1 and -1) and a model of no interaction (NI; black solid line). (H) The extent of mixing of gp41(JRFL)ΔED*-PAGFP and gp41(JRFL)ΔED*-PAmCherry, calculated for each wide area image in A.

(I) Two-colour SMLM imaging of fixed E6.1 Jurkat cells expressing TCRζ-Dronpa, stained and imaged for pTCRζ (dSTORM) on an αCD3-coated coverslips. Cells were dropped and let spread on the coverslip for 3 min before fixation. Bars – 2 μm (left) and 200 nm (right). Shown is a representative cell (N=5). Error bars are SEM.

**Fig. S6. Gp41 leads to massive cell killing and promotes CD69 upregulation**

(A) Bright-field microscopy of unstimulated Jurkat E6.1 cells, expressing gp41 constructs (either gp41(JRFL)ΔED*-YFP or gp41(HXB2)-YFP). Cells were stained with Trypan Blue to indicate cell death (dark grey-levels). (B) Fluorescence microscopy of unstimulated Jurkat E6.1 cells, expressing gp41 constructs (either gp41(JRFL)ΔED*-YFP or gp41(HXB2)-YFP). Right-most column in panel B shows enhanced brightness of the CD69-Alexa647 channel, to highlight the enhanced CD69 upregulation by the HXB2 construct. (C) Bright-field microscopy of TCR-stimulated Jurkat E6.1 cells, expressing gp41 constructs (either gp41(JRFL)-YFP or gp41(HXB2)-YFP). Cells were stained with Trypan Blue to indicate cell death (dark grey-levels). (D) Fluorescence microscopy of TCR-stimulated Jurkat E6.1 cells, expressing gp41 constructs (either gp41(JRFL)ΔED*L)-YFP or gp41(HXB2)-YFP). Left-most column shows zoomed bright-field images of individual cells for cell expressing no gp41 or gp41(JRFL)ΔED*, and cell debris for cells expressing gp41(HXB2). Scale bars – 10 μm.

**Movie M1. The recruitment of gp41 and TCRζ at early contacts of the IS**

Two-colour PALM imaging of a live Jurkat E6.1 cell expressing TCRζ-Dronpa and gp41(JRFL)ΔED*-PAmCherry on an αCD3-coated coverslips. Each of the images was collected from 50 frames at a frame rate of 13.1 fps, yielding an effective frame time of 3.8s. (see SI for further details on live cell imaging).

**Movie M2. The recruitment of gp41 and TCRζ at early contacts of the IS, after Kalman filtering**

Two-colour PALM imaging of a live Jurkat E6.1 cell expressing TCRζ-Dronpa and gp41(JRFL)ΔED*-PAmCherry on an αCD3-coated coverslips. Each of the images was collected from 50 frames at a frame rate of 13.1 fps, yielding an effective frame time of 3.8s. Shown is a reconstructed movie after Kalman filtering for accounting for the fast photobleaching of Dronpa (see SI for further details on live cell imaging and Kalman filtering).

# Table S1

| Experiment | Gp41(JRFL)ΔED* & TCRζ on αCD3 | Gp41(JRFL)ΔED*-mCRAC & TCRζ on αCD3 | Gp41(JRFL)ΔED* & TCRζ on αCD45 |
|---|---|---|---|
| **Number of cells** | 13 | 16 | 20 |
| **Footprint Area (μm²)** | 405 ±59 | 276 ±32 | 87 ±8 |
| **Gp41 (#/1000)** | 16.1 ±1.6 | 14.6 ±2.7 | 11.4 ±1.2 |
| **TCR (#/1000)** | 41.5 ±2.7 | 49 ±8.4 | 16.1 ±2.5 |

| | | | |
|---|---|---|---|
| **Area (center/surround)** | 0.23 ±0.06 | 0.38 ±0.08 | 1.1 ±0.19 |
| **Gp41 (center/surround)** | 1 ±0.20 | 1.5 ±0.59 | 2.9 ±0.57 |
| **TCR (center/surround)** | 0.38 ±0.10 | 0.39 ±0.13 | 1.47 ±0.45 |

| | | | |
|---|---|---|---|
| **Gp41 Center density (#/μm²)** | 125 ±15 | 115 ±14 | 198 ±20 |
| **Gp41 Surround density (#/μm²)** | 31 ±6 | 27 ±4 | 85 ±15 |
| **(P-value) Gp41density center vs. surround** | (3.9E-5) | (1.1E-5) | (9.4E-5) |

| | | | |
|---|---|---|---|
| **TCR Center density (#/μm²)** | 170 ±21 | 186 ±17 | 211 ±21 |
| **TCR Surround density (#/μm²)** | 111 ±16 | 163 ±15 | 166 ±18 |
| **(P-value) TCR density center vs. surround** | (.038) | NS (0.34) | NS (0.12) |

Comments:
Red text – of specific interest
NS – Not significant. Errors – SEM.

# Figure S1

A

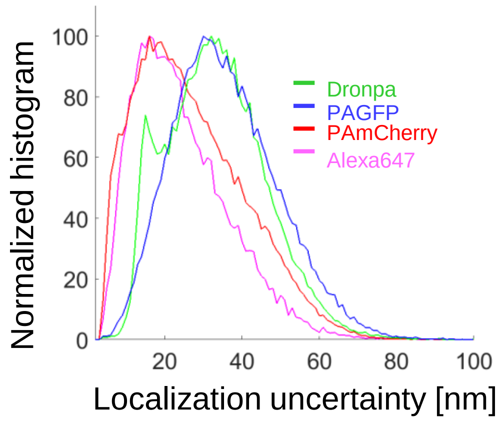


B

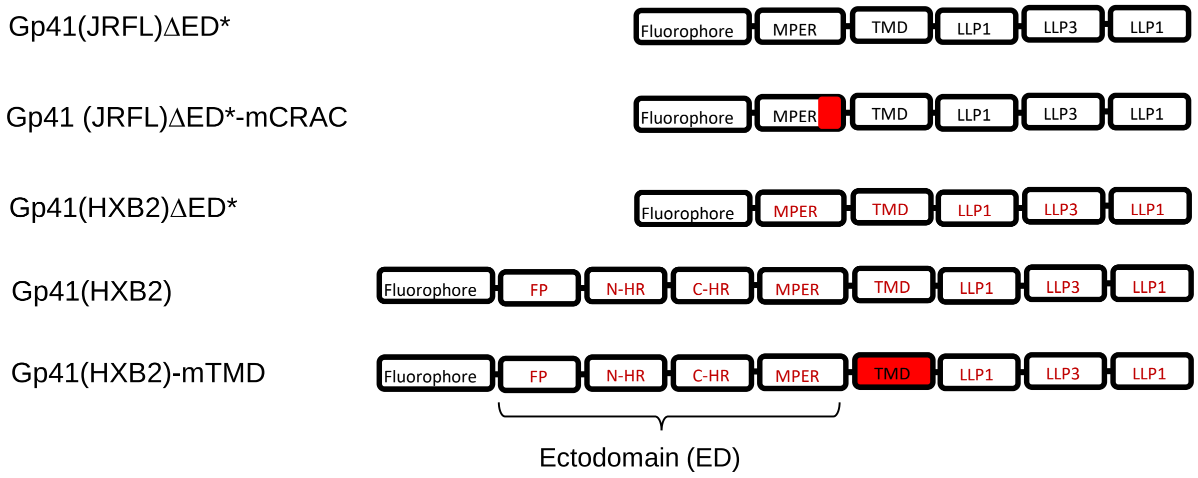


C

```
JRFL  AVGIGAVFLGFLGAAGSTMGAASMTLTVQARLLLSGIVQQQNNLLRAIEAQQRMLQLTVW
HXB2  AVGIGALFLGFLGAAGSTMGAASMTLTVQARQLLSGIVQQQNNLLRAIEAQQHLLQLTVW
      ******:************************ ********************.:******

JRFL  GIKQLQARVLAVERYLGDQQLLGIWGCSGKLICTTAVPWNASWSNKSLDRIWNNMTWMEW
HXB2  GIKQLQARILAVERYLKDQQLLGIWGCSGKLICTTAVPWNASWSNKSLEQIWNHTTWMEW
      ********:******* *******************************:.***: *****

JRFL  EREIDNYTSEIYTLIEESQNQQEKNEQELLELDKWASLWNWFDITKWLWYIKIFIMIVGG
HXB2  DREINNYTSLIHSLIEESQNQQEKNEQELLELDKWASLWNWFNITNWLWYIKLFIMIVGG
      :***:**** *::*****************************:**:******:*******

JRFL  LVGLRLVFTVLSIVNRVRQGYSPLSFQTLLPAPRGPDRPEGIEEEGGERDRDRSGRLVNG
HXB2  LVGLRIVFAVLSIVNRVRQGYSPLSFQTHLPTPRGPDRPEGIEEEGGERDRDRSIRLVNG
      *****:**:******************* **:********************** *****

JRFL  FLALIWVDLRSLCLFSYHRLRDLLLTVTRIVELLGRRGWEVLKYWWNLLQYWSQELKNSA
HXB2  SLALIWDDLRSLCLFSYHRLRDLLLIVTRIVELLGRRGWEALKYWWNLLQYWSQELKNSA
       ***** ****************** **************.*******************

JRFL  VSLLNATAIAVAEGTDRIIEALQRTYRAILHIPTRIRQGLERALL
HXB2  VSLLNATAIAVAEGTDRVIEVVQGACRAIRHIPRRIRQGLERILL
      *****************:**.:* : *** *** ******** **
```

# Figure S2

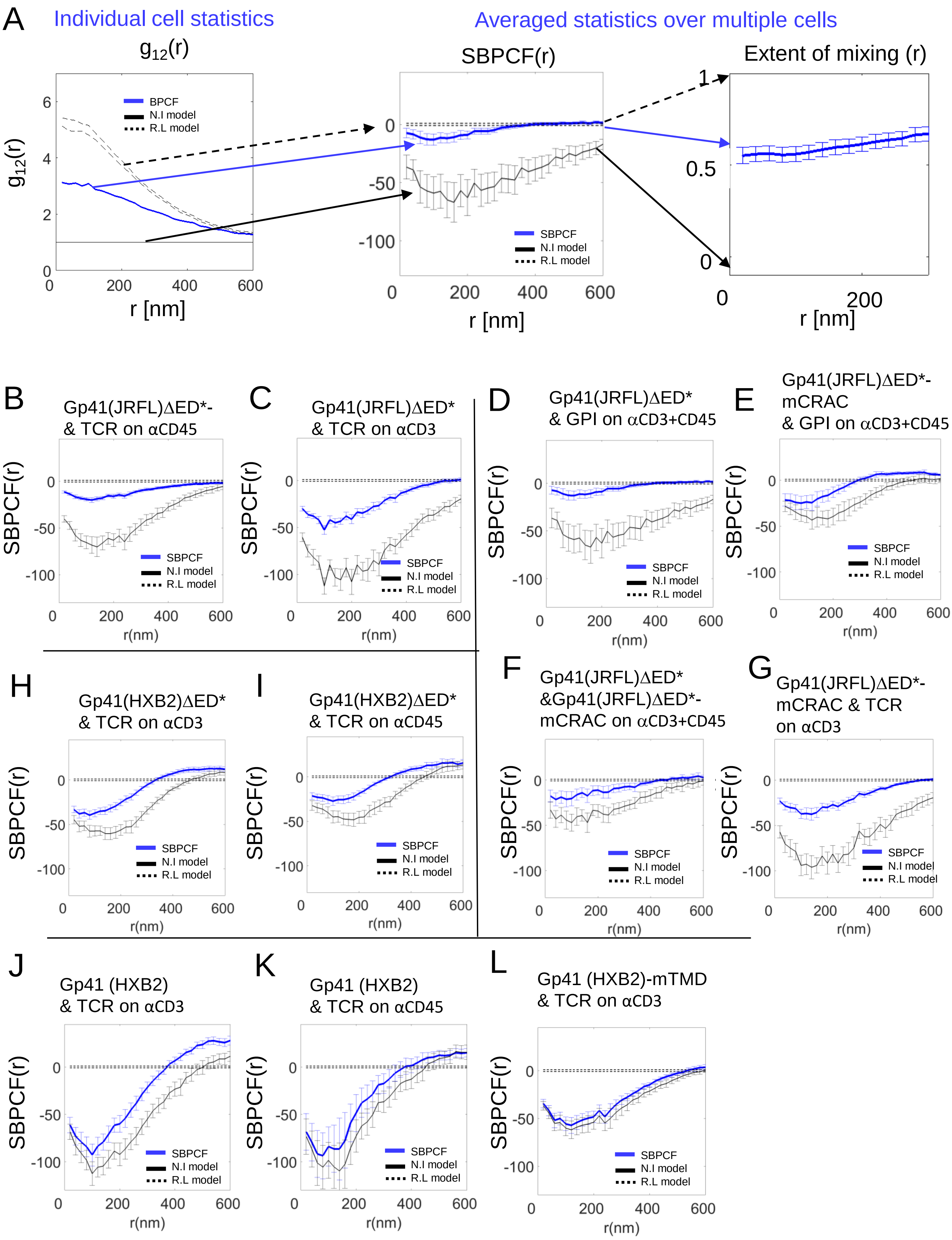
A
Individual cell statistics
Averaged statistics over multiple cells
$g_{12}(r)$
SBPCF(r)
Extent of mixing (r)
BPCF
N.I model
R.L model
SBPCF
r [nm]
B
Gp41(JRFL)ΔED*-
& TCR on αCD45
C
Gp41(JRFL)ΔED*
& TCR on αCD3
D
Gp41(JRFL)ΔED*
& GPI on αCD3+CD45
E
Gp41(JRFL)ΔED*-
mCRAC
& GPI on αCD3+CD45
F
Gp41(JRFL)ΔED*
&Gp41(JRFL)ΔED*-
mCRAC on αCD3+CD45
G
Gp41(JRFL)ΔED*-
mCRAC & TCR
on αCD3
H
Gp41(HXB2)ΔED*
& TCR on αCD3
I
Gp41(HXB2)ΔED*
& TCR on αCD45
J
Gp41 (HXB2)
& TCR on αCD3
K
Gp41 (HXB2)
& TCR on αCD45
L
Gp41 (HXB2)-mTMD
& TCR on αCD3
r(nm)

Figure S3

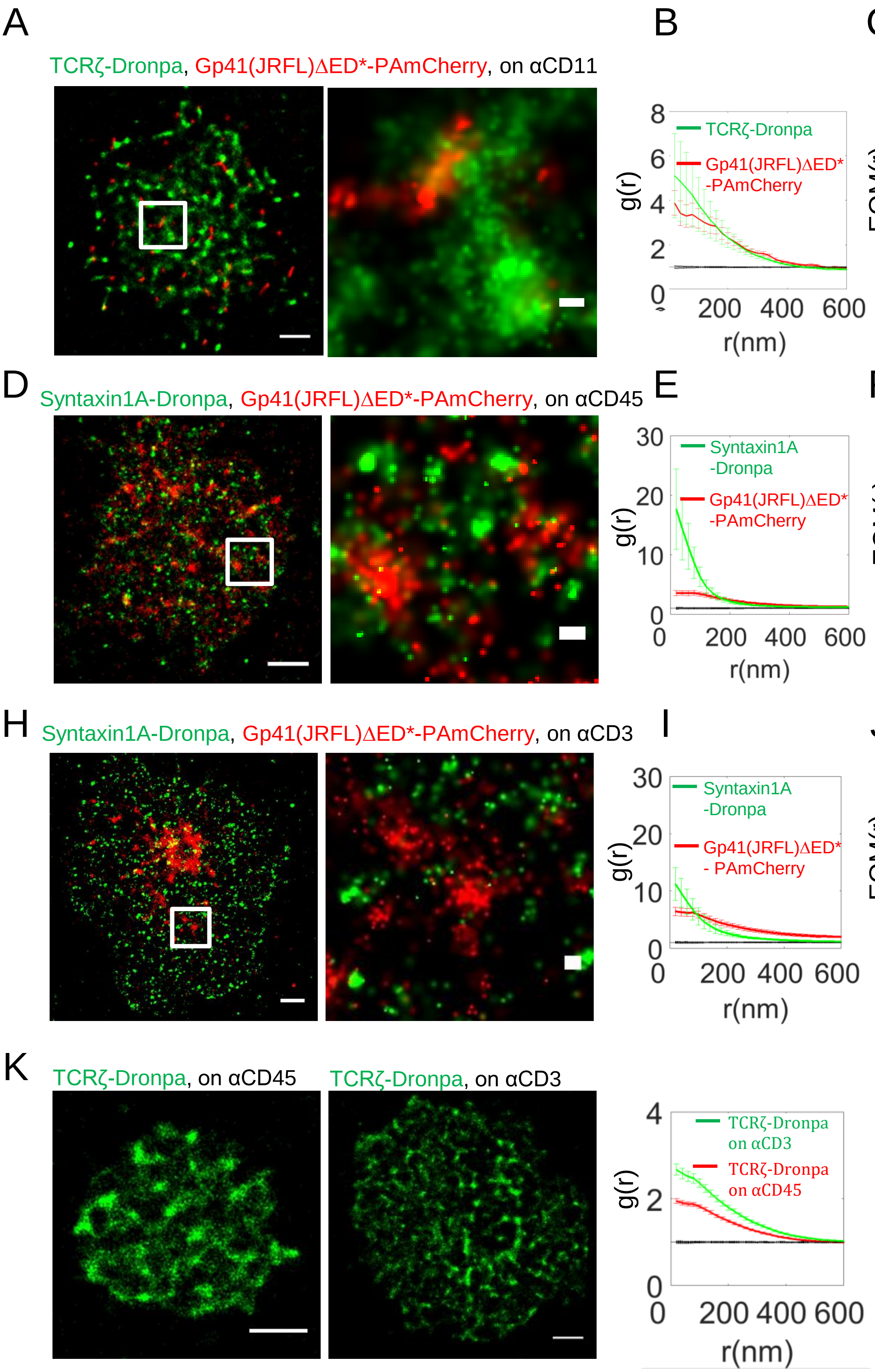

A
TCRζ-Dronpa, Gp41(JRFL)ΔED*-PAmCherry, on αCD11
B
TCRζ-Dronpa
Gp41(JRFL)ΔED*
-PAmCherry
g(r)
r(nm)
C
EOM(r)
r(nm)
D
Syntaxin1A-Dronpa, Gp41(JRFL)ΔED*-PAmCherry, on αCD45
E
Syntaxin1A
-Dronpa
Gp41(JRFL)ΔED*
-PAmCherry
g(r)
r(nm)
F
EOM(r)
r(nm)
H
Syntaxin1A-Dronpa, Gp41(JRFL)ΔED*-PAmCherry, on αCD3
I
Syntaxin1A
-Dronpa
Gp41(JRFL)ΔED*
- PAmCherry
g(r)
r(nm)
J
EOM(r)
r(nm)
K
TCRζ-Dronpa, on αCD45
TCRζ-Dronpa, on αCD3
TCRζ-Dronpa
on αCD3
TCRζ-Dronpa
on αCD45
g(r)
r(nm)

# Figure S4

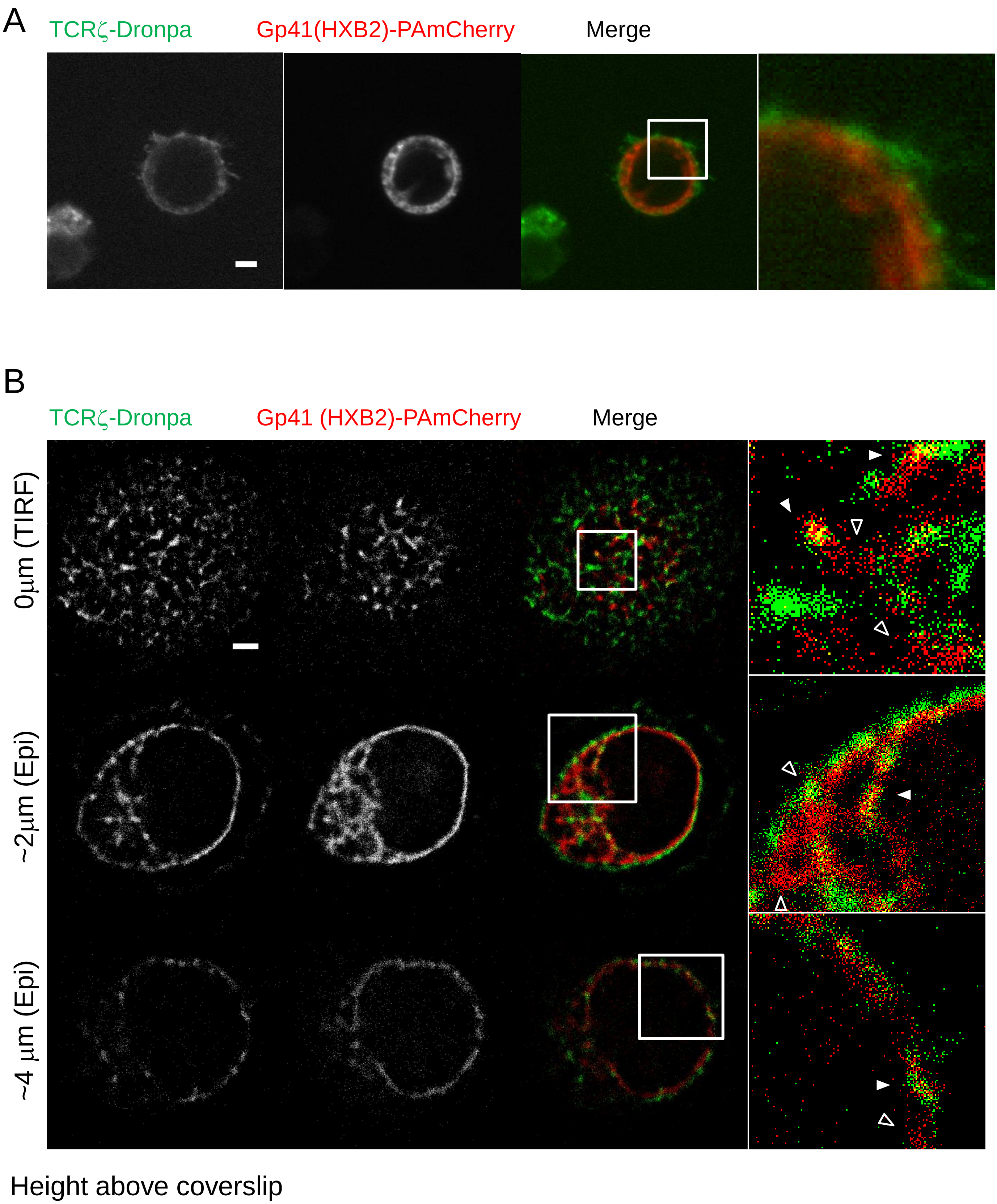
A
TCRζ-Dronpa
Gp41(HXB2)-PAmCherry
Merge
B
TCRζ-Dronpa
Gp41 (HXB2)-PAmCherry
Merge
0μm (TIRF)
~2μm (Epi)
~4 μm (Epi)
Height above coverslip

# Figure S5

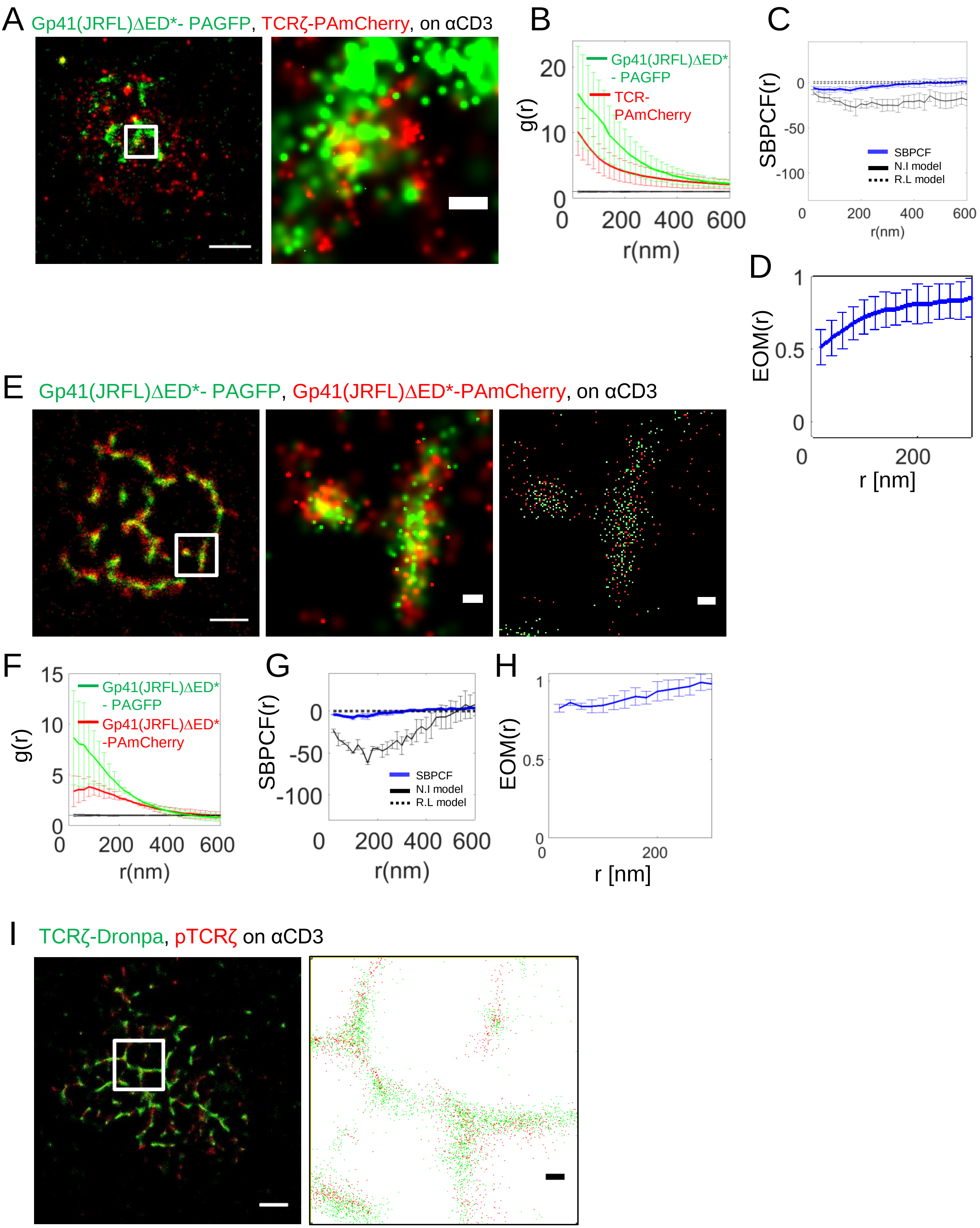
A Gp41(JRFL)ΔED*- PAGFP, TCRζ-PAmCherry, on αCD3
B
Gp41(JRFL)ΔED* - PAGFP
TCR-PAmCherry
g(r)
r(nm)
C
SBPCF(r)
SBPCF
N.I model
R.L model
r(nm)
D
EOM(r)
r [nm]
E Gp41(JRFL)ΔED*- PAGFP, Gp41(JRFL)ΔED*-PAmCherry, on αCD3
F
Gp41(JRFL)ΔED* - PAGFP
Gp41(JRFL)ΔED* -PAmCherry
g(r)
r(nm)
G
SBPCF(r)
SBPCF
N.I model
R.L model
r(nm)
H
EOM(r)
r [nm]
I TCRζ-Dronpa, pTCRζ on αCD3

# Figure S6

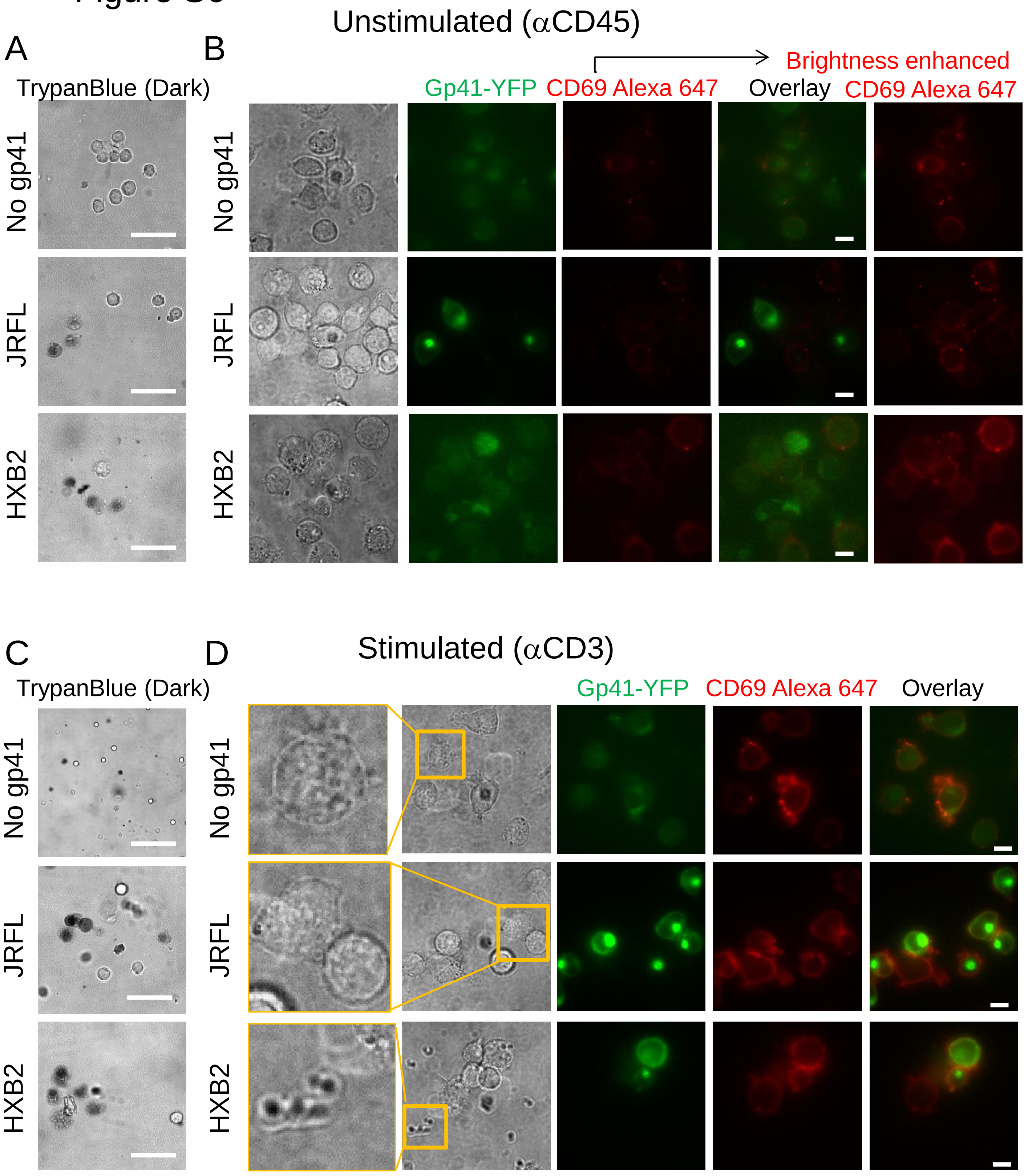

A
B
Unstimulated (αCD45)
TrypanBlue (Dark)
Brightness enhanced
Gp41-YFP
CD69 Alexa 647
Overlay
CD69 Alexa 647
No gp41
JRFL
HXB2
C
D
Stimulated (αCD3)
TrypanBlue (Dark)
Gp41-YFP
CD69 Alexa 647
Overlay
No gp41
JRFL
HXB2

# Figure S7

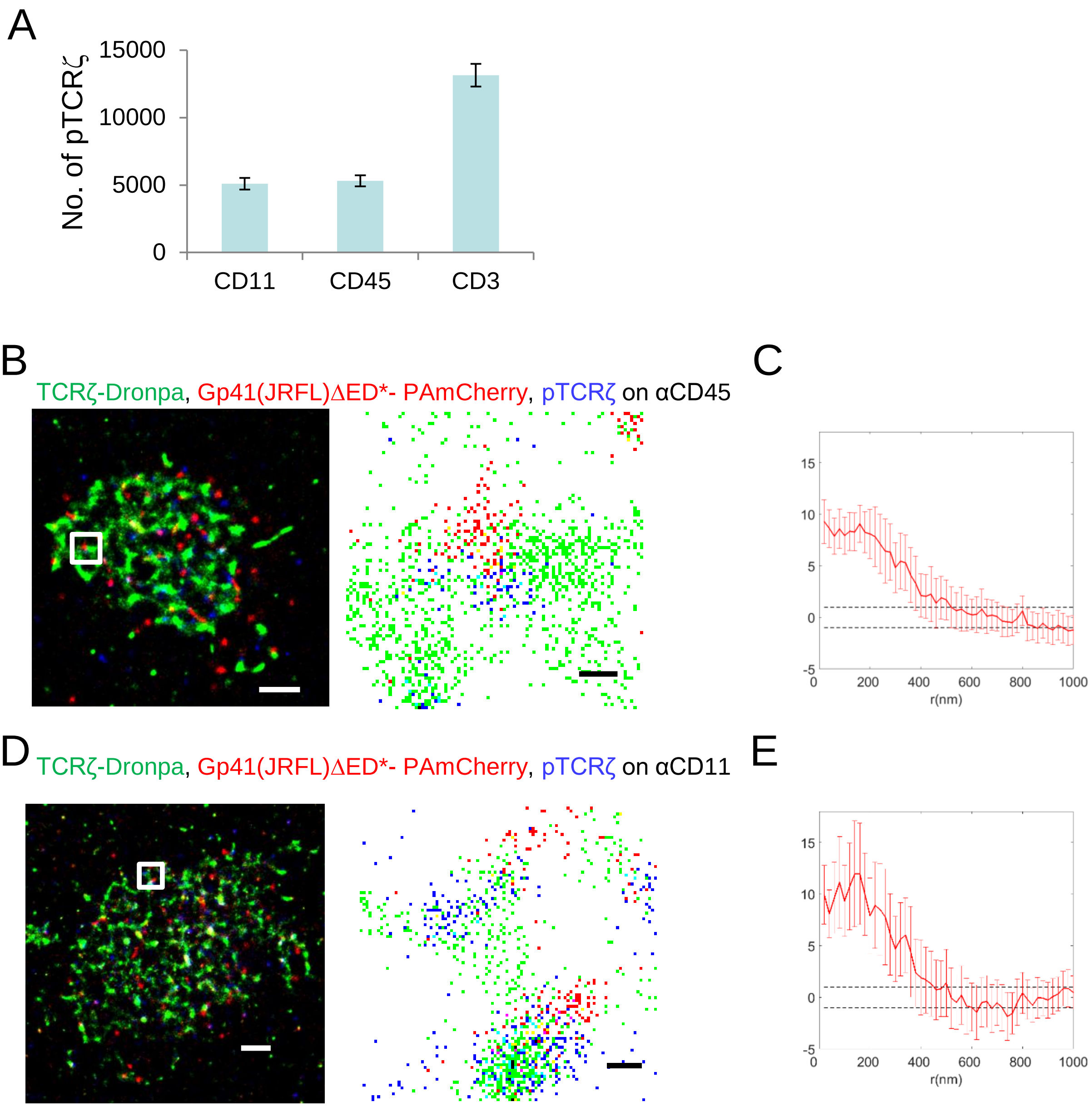
A
No. of pTCRζ
15000
10000
5000
0
CD11
CD45
CD3
B
TCRζ-Dronpa, Gp41(JRFL)ΔED*- PAmCherry, pTCRζ on αCD45
C
15
10
5
0
-5
0
200
400
600
800
1000
r(nm)
D
TCRζ-Dronpa, Gp41(JRFL)ΔED*- PAmCherry, pTCRζ on αCD11
E
r(nm)

## Movie M1

TCRζ-Dronpa, Gp41(JRFL)ΔED*-PAmCherry on αCD3

Reconstructed PALM

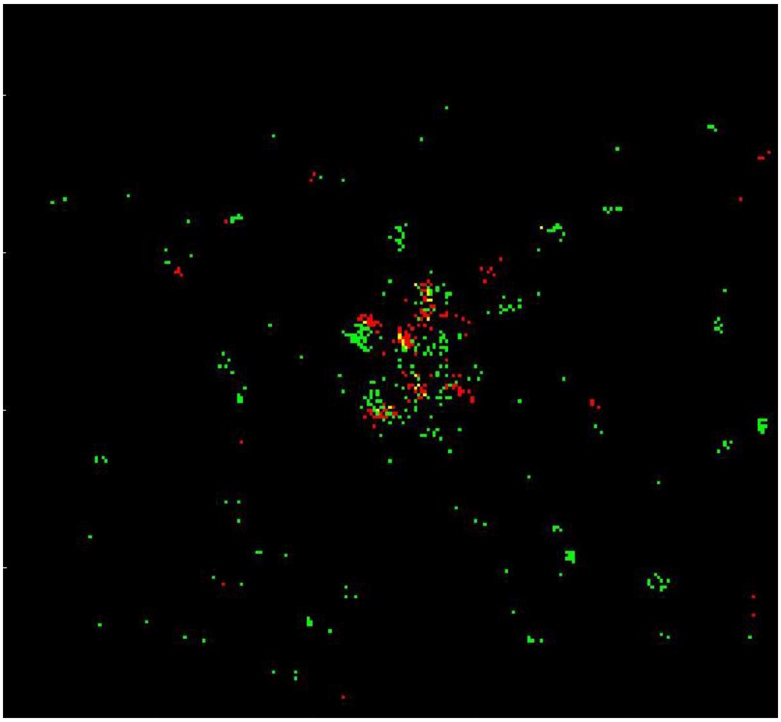

## Movie M2

TCRζ-Dronpa, Gp41(JRFL)ΔED*-PAmCherry on αCD3

Reconstructed PALM after Kalman filtering

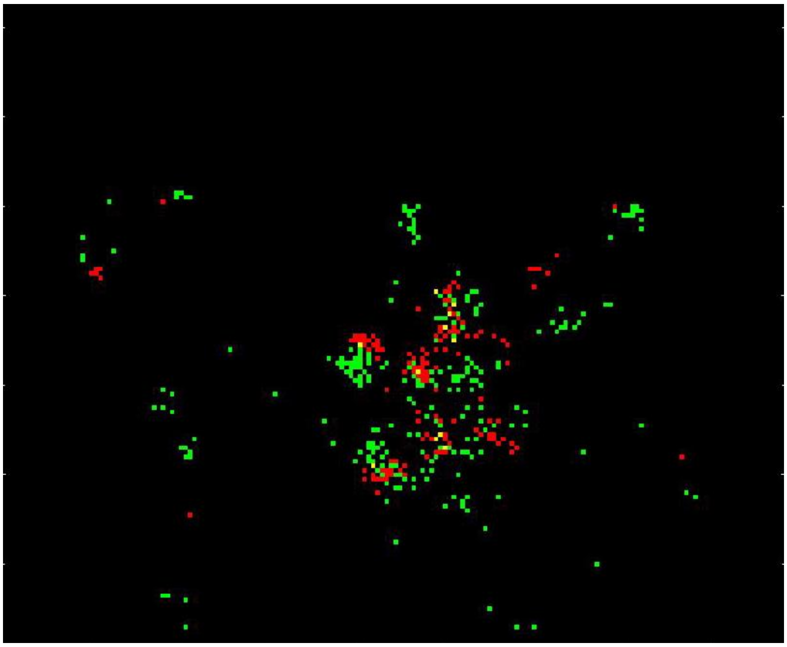

Supplement: Supplementary file 1 — Supplementary Information [file 41598_2018_28114_MOESM1_ESM.pdf]
